# Prioritising Conservation Area in Species Management Strategy for The Edible Bornean Giant River Frog *Limnonectes leporinus* Anderson 1923

**DOI:** 10.21315/tlsr2024.35.3.3

**Published:** 2024-10-07

**Authors:** Ramlah Zainudin, Elvy Quatrin Deka, Julius Georgy

**Affiliations:** 1 Molecular Ecology Lab, Faculty of Resource Science and Technology, Universiti Malaysia Sarawak, 94300 Kota Samarahan, Sarawak, Malaysia; 2 Real Living Laboratory, Faculty of Resource Science and Technology, Universiti Malaysia Sarawak, 94300 Kota Samarahan, Sarawak, Malaysia

**Keywords:** Landscape Fragmentation, Spatial Modelling, Habitat Connectivity, Gene Flow, Species Distribution, Pemecahan Landskap, Pemodelan Spatial, Keterkaitan Habitat, Aliran Gen, Taburan Spesies

## Abstract

*Limnonectes leporinus*, the endemic giant river frog, is a riparian (stream dwelling) species that lives along streams with moderate to steep gradients. The most serious threats to the species are deforestation caused by severe clear cutting, which fragments its distribution, and overhunting for local consumption. Excessive landscape modification alters habitat, making it critical for an organism to maintain heterozygozity for the population to be fit to adapt to a changing environment. The goal of this research was to project suitable habitats and predict the potential for habitat connectivity to allow gene flow across the Sarawak landscape. The presence data file and environmental layers were converted into ASCII format using ArcGIS and then used in MaxEnt modelling to generate the map of suitable habitats. To perform the connectivity model, the potential habitat model and genetic attributes of haplotype data were computerised in Circuitscape software. The findings revealed that suitable habitats corresponded to species distribution in lowland areas with sustainable stream networks as breeding sites, while higher elevations were identified as unsuitable habitats. Gene flow data, on the other hand, revealed that connectivity circuits are mostly found in unprotected forest, which includes development areas and private lands. As a result, prioritising conservation areas should include local governments and landowners in proper landscape management as well as species management strategies. This indirectly sustains and protects Borneo’s forests, flora and fauna.

## Abstract (fr)

**Abstrak:**

*Limnonectes leporinus*, katak sungai gergasi endemik, ialah spesies riparian (mendiami sungai) yang hidup di sepanjang sungai dengan kecerunan sederhana hingga curam. Ancaman yang paling serius terhadap spesies ini ialah penebangan hutan yang teruk, yang memecah-belah taburan dan pemburuan berlebihan makanan tempatan. Pengubahsuaian landskap yang berlebihan mengubah habitat, menjadikannya kritikal bagi organisma untuk mengekalkan heterozigoziti agar populasi sesuai untuk menyesuaikan diri dengan persekitaran yang sentiasa berubah. Matlamat penyelidikan ini adalah untuk mengenal pasti habitat yang sesuai dan meramalkan potensi kesinambungan habitat untuk membolehkan aliran gen merentasi landskap Sarawak. Fail data kehadiran dan lapisan persekitaran ditukar kepada format ASCII menggunakan ArcGIS dan kemudian digunakan dalam pemodelan MaxEnt untuk menjana peta habitat yang sesuai. Untuk melaksanakan model ketersambungan, model habitat berpotensi dan sifat genetik data haplotaip telah dikomputerkan dalam perisian Circuitscape. Hasil kajian mendedahkan bahawa habitat yang sesuai sepadan dengan taburan species di kawasan tanah pamah dengan rangkaian sungai yang mampan sebagai tapak pembiakan, manakala kawasan yang lebih tinggi dikenal pasti sebagai habitat yang tidak sesuai. Data aliran gen, sebaliknya, mendedahkan bahawa litar penyambungan taburan kebanyakannya ditemui di hutan tidak dilindungi, yang merangkumi kawasan pembangunan dan tanah persendirian. Akibatnya, keutamaan kawasan pemuliharaan harus merangkumi kerajaan tempatan dan pemilik tanah dalam pengurusan landskap yang betul serta strategi pengurusan spesies. Ini secara tidak langsung mengekalkan dan melindungi hutan, flora dan fauna Borneo.

**Highlights**
- Created a map of acceptable habitat for anuran species using three factors (species distribution, genetic data and microhabitat utilisation). The data used is the result of interactions between species and their biotic (gene flow) and abiotic (microhabitats and temperature) elements, which are critical for species survival and do not rely exclusively on the ability to adapt to climate change (temperature).
- Suitable habitats corresponded to species distribution in lowland areas with sustainable stream networks as breeding sites, while higher elevations were identified as unsuitable habitats.
- Gene flow data revealed that connectivity circuits are mostly found in unprotected forest, which includes development areas and private lands.
- Prioritising conservation areas should include local governments and landowners in proper landscape management as well as species management strategies.

## INTRODUCTION

The island of Borneo is well known as a Southeast Asian hotspot for biodiversity: biologically rich but deeply threatened. This is a global priority for conservation due to high levels of diversity and endemism along with a high level of threats. Conservation planning is essential to ensure that hotspots of biodiversity and endemism have the protection needed to prevent deforestation, hunting and overexploitation in their most diverse areas, but these data are still lacking for this region. Furthermore Collen *et al*. (2008) have addressed on the issue on disparity in global monitoring of tropical biodiversity. Alarmed by the current situation occurring in Sarawak, a state in Malaysian Borneo, one endemic species of fanged frog was taken into consideration as a species model to assess further its ability to disperse throughout patches of degraded natural habitat of the extant populations.

*Limnonectes leporinus*, also commonly known as the giant river frog, was widely hunted for the thigh flesh, which is edible by the local community (Iskandar 2004; Ho *et al*. 2008). This species is widely distributed throughout Sarawak (Inger *et al*. 2017) and is listed as of Least Concern in The International Union for Conservation of Nature’s (IUCN) Red List (IUCN 2021). Grafe (2011) found that *L. leporinus* are strictly riparian spending most of their time in restricted core activity areas between 450 m^2^–1,910 m^2^ within 20 m from the stream bed. Both males and females performed long distance migrations associated with reproductive activity within the stream. Home range sizes ranged between 2,050 m^2^–8,250 m^2^. Trakhtenbrot *et al*. (2005) stated that long-distance dispersal events (especially in highly dispersed species) are crucial to population spread and to the maintenance of genetic connectivity. Despite being widespread in lowland primary forests, *L. leporinus* is an excellent candidate for studying species connectivity because it may be experiencing population losses due to its limited habitat tolerances and migratory behaviour (Grafe 2011).

The acceleration of deforestation and land clearance activities for rapid development throughout Borneo has led to a major problem for *L. leporinus*, i.e., its habitat loss. Fragmentation of habitats may lead to genetic break due to disruption of gene flow among populations. Thus, the aims of this study were to identify suitable habitats and to indicate viable landscape connectivity to aid the gene flow of the extant populations of *L. leporinus* through model projections.

The maximum entropy (MaxEnt) approach for spatial modelling, as described by Phillips and Dudik (2008), only requires presence data in cases when absence data are not readily accessible. Nonetheless, this approach is an ideal tool since it yielded most reliable predictions by producing more accurate outputs from small numbers of data points (Merow *et al*. 2013). Circuitscape was employed by applying circuit theory to allow gene flow across resistant surfaces through random movement in every direction (McRae *et al*. 2008). This study focused on prioritising conservation efforts at important areas that are identified to sustain the extant populations of *L. leporinus* in the wild.

## MATERIALS AND METHODS

### Study Areas

**Figure 1:**
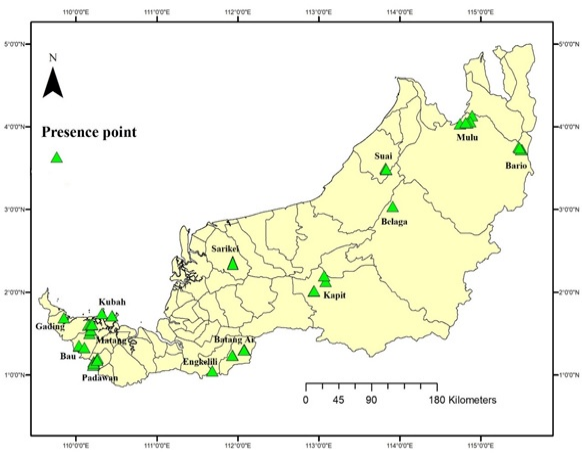
Presence data of *Limnonectes leporinus* in Sarawak for years 2000–2020 (based on UNIMAS Zoological Records). *Note*: Image is from the UNIMAS Museum Collections Data owned by author RZ.

A database of species occurrence consisting of total 90 presence points was a compilation of data collection from both field samplings at various study sites throughout Sarawak conducted from 2010 to 2020. Data of year 2000 and below (recorded in UNIMAS Zoological Museum database) was also used for species distribution. Study areas included Mulu (4° 2′ N 114° 55′ E), Bario (N 3° 45′ N 115° 27′ E), Suai (3° 29.744′ N 113° 49.892′ E), Belaga (3° 2′ N 100° 54′ E), Kapit (2° 10.139′ N 113° 03.104′ E), Batang Ai (1° 33.22′ N 111° 57.32′ E), Engkelili (1° 3′ N 111° 41′ E), Padawan (1° 8.03′ N 110° 13.55′ E), Gading (1° 41.497′ N 109° 50.767′ E), Kuching (1° 36.51′ N 110° 9.61′ E) and Bau (1° 25′ N 110° 9′ E). The locality of each sample was validated geographically, and the geographic coordinate of each sample was georeferenced in a Geographic Information System (GIS) tool (ArcGIS 10.3), as shown in Fig. 1. The surveyed areas in this study consisted of gazetted national parks (Mulu National Park, Batang Ai National Park, Kubah National Park, Gading National Park and Matang Wildlife Centre in Kuching Division) as totally protected areas, local settlements in rural areas and two sites in oil palm plantations [Wilmar Oil Palm Plantation at Suai and Malaysian Palm Oil Board (MPOB) at Sungai Asap, Belaga].

### Data Analyses

#### Habitat

There are four variable characteristics of habitats and microhabitats: vegetation type, horizontal position, vertical position and substrate (Zainudin *et al*. 2017; Zulkefli & Zainudin 2022). A principal component analysis was employed to determine the meaningful variables for the species’ microhabitats. Further analysis with non-metric dimensional scale (NMDS) was used to determine microhabitat utilisation based on the most favourable characteristics. The highest loading microhabitats were selected for the raster data map (Table 1).

#### Molecular/genetic data

Haplotypes and gene flow were analysed using Zainudin and Naim’s (2018) approaches. A population genetic analysis was carried out with molecular data to show haplotype diversity. Shared haplotypes reflect a high gene flow and connected localities, while a high population subdivision decreases the gene flow and produces a panmictic population. The haplotype sequences were then used to construct a phylogenetic tree and were categorised for the raster data map (Table 1).

#### Model predictor variables

Predictor variables were selected to estimate the models based on the species’ ecological needs and possible threats. In the natural habitat, *L. leporinus* would be found abundantly in riverine forests especially during the mating period, where gravid females are normally found depositing fertilised eggs under the rocks of gravel-bottom streams (Emerson 1992). When it senses threats to life from predators, this species uses its strong muscular hind legs to escape by jumping into nearby water bodies and swimming away for survival. This natural predator-escape reaction corresponds to the species’ preferential microhabitat selection of riverbank slopes while feeding.

Adult individuals can be detected during the non-mating season wandering on the forest floor 10 m to 20 m away from the stream, indicating that the species is strictly stream-dependent; thus, the stream network is relatively important for the dispersal of this species during migration (Inger & Voris 2001; Grafe 2011). However, the migration of the species is potentially vulnerable due to the increasing conflicts resulting from changing landscapes in the lowland areas of Sarawak (Grafe 2011). Frog species of medium-to-large body size are likely to show less movement, although *L. leporinus* could move further throughout lower elevations (Inger & Voris 2001; Inger 2009). Degraded forest areas and areas of reduced intact forest subsequently alter the environmental temperature, which could lead to desiccation of the frogs’ skin. Land clearance activities have also indirectly permitted the human population to dominate the natural habitats of many animal species, despite *L. leporinus* not being a commensal species. All these important aspects of the species’ requirements were taken into consideration to estimate the suitable spatial habitat and connectivity for *L. leporinus* in Sarawak. Seven predictor variables were included in this study: elevation, intact forest, land cover, major basin, human population density, slope and temperature.

### Modelling using MaxEnt and Circuitscape

The geographical coordinates of species’ occurrence data were recorded in an Excel file saved in the .csv file format. Prior to running MaxEnt (version 3.1; http://www.cs.princeton.edu/~schapire/maxent/; Phillips *et al*. 2017), all predictor variables were converted to raster dataset with the Extent environment for the same cell size for all layers. The raster datasets were converted to ASCII format computed in ArcGIS 10.3 software (ver. 10.3; ESRI, Redlands, CA, US; Scheldeman & van Zonneveld 2010) because this format is compatible with data computation in MaxEnt.

The species occurrence file was executed as an input file with the predictor variables as model input for environmental data in MaxEnt. The credibility of MaxEnt in determining habitat suitability was based on response curves and the jackknife method. The MaxEnt result was interpreted based on a fraction score of scale 0 indicating an area unsuitable as a species habitat to scale 1 as the best area suitable for habitat. The output ASCII file generated in MaxEnt was used in Circuitscape as a resistance map to predict the habitat connectivity of *L. leporinus* in Sarawak. According to McRae *et al*. (2008), utilisation of this map as a resistance surface to predict connectivity is reliable because it is based on a species’ probability of moving across the area of interest depending on its ecological needs.

The genetic attributes of haplotype data were prepared to perform the connectivity model in Circuitscape. The haplotypes were generated in DNASP version 6.12.01 (Universitat De Barcelona, Spain) (Rozas *et al*. 2017); the DNA sequences are available in the GenBank with accession number HQ 283101–283148 and KC 139382–139396. In the Excel file, the species name column was named NodeID, consisting of the haplotypes. The second and third columns were labelled as N for latitude and E for longitude in decimal degrees (dd) format. Individuals of populations that shared same haplotype would have same node ID. The Excel file was saved in.csv format to be converted to ASCII format using MaxEnt as focal point. Circuitscape was run based on circuit theory implying random movement or electricity current across the circuit resistance surface (McRae *et al*. 2008).

## RESULTS

The attributes of mitochondrial DNA variations (molecular DNA), and ecological data of *L. leporinus* produce the same outcomes as the horned frogs *Pelobatrachus nasutus* (Zainudin *et al*. 2023) in successfully determine the status of the organism in the fragmented habitat.

**Figure 2:**
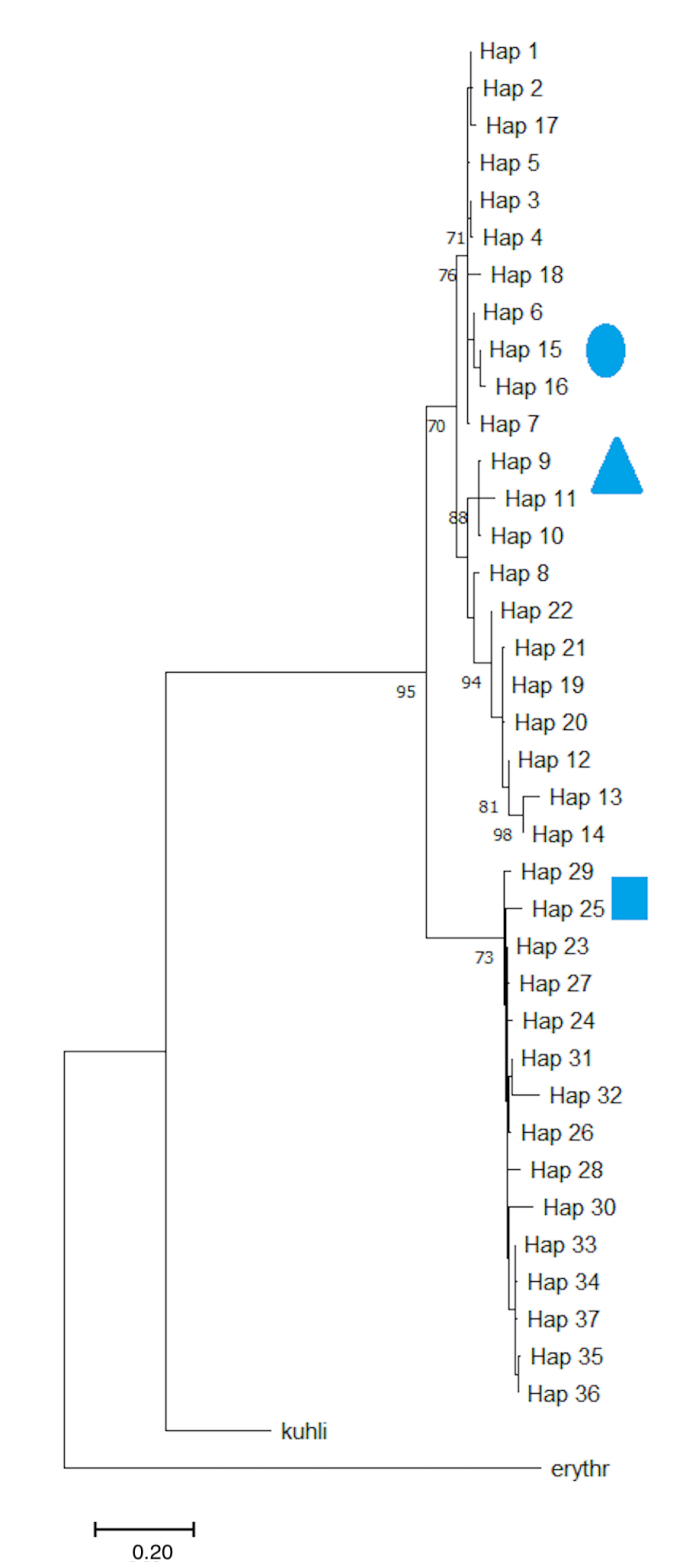
Maximum likelihood of mtDNA cytochrome oxidase 1 gene of *L. leporinu*s haplotypes across Sarawak populations. *Note*: Circle denotes Western populations, triangle denotes Central populations, and square denotes Northeastern populations. Image is from the UNIMAS Museum Collections Data owned by author RZ.

The jackknife test revealed that land cover and annual temperature were key factors which influenced the ground riverbank dweller *L. leporinus*’ habitat preferences. The data on haplotypes also revealed a high level of gene flow among *L. leporinus* populations, with the Central and Northeast Sarawak populations yielding the highest amount (Table 2) but also being the least diverge populations (Table 3). Shared haplotypes were apparent among Western populations of *L. leporinus*, suggesting high connectivity between them (Table 4). Phylogenetically, two moderately significant clades were discovered among the *L. leporinus* lineages (Fig. 2), namely Western-Central [70% maximum likelihood (ML)], and Northeast clades (76% ML). This implies a genetic break among the clades, an indicator of disruptive connectivity of *L. leporinus* among the Sarawak populations.

### Identification of Suitable Habitat Areas for *L. leporinus*

**Figure 3:**
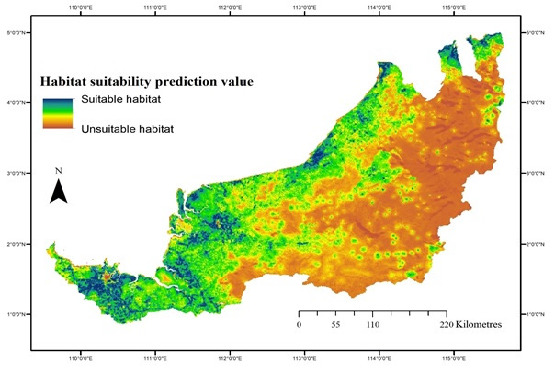
Map of suitable habitat for *L. leporinus* in Sarawak, generated in MaxEnt. *Note*: Image is from the UNIMAS Museum Collections Data owned by author RZ.

The MaxEnt approach identified potential suitable habitat areas for *L. leporinus* in Sarawak. The predicted model in Fig. 3 defined areas of potential species habitat based on ecological requirements. In Fig. 3, the model predicted highland areas in Sarawak as unsuitable habitat for *L. leporinus*, corresponding to the distribution of this species in lowland areas where stream networks are present for breeding sites. Although the suitable areas may provide breeding sites, one of most important aspect to sustain the species, the suitable habitats are, however, heavily developed areas with dense human populations. Therefore, predicting suitable habitat is essential in the actual mapping of species distribution for conservation prioritisation.

### Connectivity Model for Conservation Strategy

**Figure 4:**
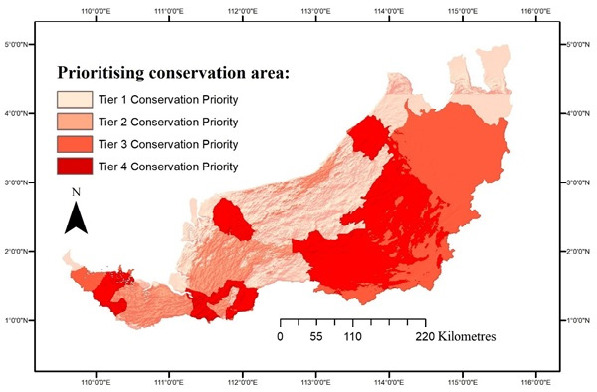
Priority areas for conservation efforts in species management strategy. *Note*: Image is from the UNIMAS Museum Collections Data owned by author RZ.

A tiered approach was employed to classify areas according to level of priority for conservation. Poor *et al*. (2012) showed that this tiered approach manages to give useful insights into conservation strategy in the study areas. Areas in Tier 1 (Fig. 4) showed the least connected areas, probably due to habitat fragmentation created by landscape being heavily modified for infrastructure projects such highways. Hence, this finding suggested that conservation in areas under Tier 1 should be carried out by focusing appropriately on the planning authority. This is critical in reducing the massive impact of anthropogenic activities in damaging further the remaining or nearby remnants where the species is present. Altered landscapes may reduce or eliminate the ecological resources for species survival; consequently, it may affect adaptation traits and lead to difficulties in reproduction and reduced fitness for migration. It is crucial to understand that lack of habitat connectivity may cause genetic breaks and result in no or limited gene flow and thus forming isolated populations. Conserving areas in Tier 1 is important; in addition, *L. leporinus* inhabits and disperses throughout lowland areas. This study postulates if these Tier 1 areas are successfully managed, there will be several small, isolated populations in fragmented habitats rather than a few large connected populations.

Tier 2 demonstrated habitat connectivity, implying a gene flow from west to east that eventually connected with the northern part of Sarawak. The connected habitat circuit falls largely in unprotected forest, which requires intensive efforts between the local authority and landowners. Similar findings in another study also showed that potential areas of high connectivity were located on private land (Pilliod *et al*. 2015). In this case, the potential of connecting habitats of *L. leporinus* on private land also means that the populations in these areas are facing threats of over-harvesting for consumption of leg meat and habitat loss. According to Ho *et al*. (2008), frog meat is favoured over chicken meat due to its rich protein and relatively low-fat content. Over-harvesting of frog species for meat to supply the high demands of the marketplace has seen an increasing trend to over-exploit the wild population, in addition to providing income for local harvesters (Kusrini 2005). Although Grafe (2011) suggested that harvesting *L. leporinus* for its meat does not seem to affect the population in the wild, conservation efforts in these areas should still include raising public awareness in the local community to protect the area. Authorities are responsible for reaching agreement with the local community to protect important areas by proposing certain areas to be gazetted as protected by the establishment of new national parks or sanctuaries.

Tier 3 showed moderate connectivity potential where it covered areas predicted as unsuitable habitat for *L. leporinus*, especially in the northern region, emphasising that priority conservation areas should focus on those overlapping with Tier 4, as that tier contains the extant populations, implying that the conservation strategy for these areas should focus on sustaining the viable populations and maintaining healthy traits to ensure that individuals are able to breed and migrate across remnants.

## DISCUSSION

The prediction shows the possibility of gene flow in areas where the presence of geographical barriers to the dispersal of frog species across Sarawak, such as the Lupar Gap (Zainudin *et al*. 2010), is suggested as a factor reducing gene flow and subdividing the populations of *L. leporinus* from the West to the Central and Northeastern of Sarawak (Zainudin 1998). However, frogs are intolerant to saltwater (Hadil 2002) indicating that the predicted gene flow from west to north across the Lupar Gap may be facilitated by overland movement of the frogs at Kalimantan. Overall, the predicted habitat connectivity for *L. leporinus* in Sarawak, as shown in Fig. 3, shows the potential for continuous connectivity allowing gene flow from the Western to the Northern parts of Sarawak. Prioritising conservation areas would be very useful in managing the important areas and thus could facilitate the migration of *L. leporinus* and preventing it from local extinction. This is further supported by Grafe (2011) in which he suggested that narrow habitat tolerance and migratory activity makes *L. leporinus* vulnerable to population decline. Furthermore, Matsui *et al*. (2015) found that mtDNA phylogeny of 12S–16S rRNA genes indicates the species not markedly diversified within the island, conforming to the fact that it is also not diversified morphologically. The divergence times among local samples are thought to be younger than some congeneric species, and it is estimated that the species arose relatively new and subsequently rapidly dispersed within the island.

The results also suggested that MaxEnt performs well with small sample record numbers as in Escalante *et al*. (2013). The finding indicates that most non-protected areas fall into the projection model. Thus, the conservation strategy should consider these areas, and further efforts involving the local authority and community are needed to ensure that the areas are protected. Anthropogenic impacts may also be taken into consideration while managing the areas and raising public awareness of the importance of biodiversity in balancing the ecosystem. More protected areas should be established for a wide variety of reasons, not only reflecting biodiversity status and endemism, but McCreless *et al*. (2013) also includes the involvement of social, political, and historical drivers. Otherwise, species and systems may be left with low levels of representation inside protected areas and potentially vulnerable to habitat loss or degradation, hunting or other drivers of species loss (Hughes 2017).

## CONCLUSION

Protecting places indicated as hyper-important in the models is a straightforward technique to ensure that biodiversity hotspots are effectively protected. It is feasible to acquire a far greater understanding of landscape connectivity patterns by combining species distribution data with the necessary environmental data than by relying alone on IUCN mapping. Finally, *L. leporinus* could be utilised as an indicator for the distribution pattern of other frog species with similar ecological needs not just in the local region, but also globally, where habitat alteration is at its peak.

## Supplementary Information



## Figures and Tables

**Table 1:** Raster data map of *Limnonectes leporinus* haplotype computed in Circuitscape.

Sample ID	Field ID	Haplotype ID	Decimal_latitude	Decimal_longitude	Clade group	Microhabitats code*			

						Vegetation	Horizontal position	Vertical position	Substrate
Ma10	MWC04	1	1.611917	110.1603	Western	11	16	43	60
Ku01	RZ113	1	1.604117	110.1858	Western	6	17	43	60
Ku02	RZ124	1	1.61115	110.1972	Western	6	17	43	60
Ku03	RZ102	1	1.610883	110.1972	Western	6	17	43	60
Ma06	RZ208	1	1.612067	110.1605	Western	11	16	43	59
Ma09	1245	1	1.612	110.1604	Western	11	16	43	59
Ma12	1239	1	1.611833	110.1602	Western	11	16	43	59
Ma11	1242	1	1.612317	110.1608	Western	11	16	43	59
Ba05	1207	1	1.367083	110.1688	Western	11	18	41	59
Ba01	1212	1	1.3672	110.1691	Western	11	18	41	59
Ba04	1214	1	1.36715	110.1689	Western	11	18	41	59
Ba06	1215	1	1.383517	110.1688	Western	11	18	41	59
Ma08	1236	1	1.61225	110.1607	Western	11	16	43	59
Ba02	1204	1	1.367167	110.169	Western	11	18	42	52
Ga03	GNP108	1	1.611083	110.1639	Western	6	16	43	60
Ga01	GNP84	1	1.611217	110.1654	Western	6	16	41	59
Ma13	MWC15	2	1.611483	110.1635	Western	11	16	41	59
Pa02	KK	3	1.133717	110.2241	Western	6	18	42	52
Pa01	OO	4	1.133833	110.2258	Western	6	18	41	59
Ma07	MWC07	5	1.61215	110.1606	Western	11	16	43	59
Ba03	1211	6	1.367317	110.1691	Western	11	17	43	60
Ma05	MWC11	7	1.611567	110.1626	Western	11	16	43	59
Ku04	KNP10	8	1.611617	110.1976	Western	6	18	46	53
Ma15	1233	9	1.611	110.164	Western	11	18	46	53
Ku05	RZ103	9	1.61165	110.1627	Western	6	18	40	59
Pa03	REGU01	9	1.611717	110.1976	Western	6	18	43	60
Ga02	GNP131	9	1.11825	110.2085	Western	6	18	40	59
Ba07	1213	10	1.383683	110.1688	Western	11	18	40	59
Pa04	UU	11	1.118367	110.211	Western	6	18	40	59
Pa05	NN	12	1.133517	110.2227	Western	6	18	40	59
Pa06	QQ	13	1.133417	110.2227	Western	6	17	43	60
Ma02	MWC16	14	1.6112	110.1637	Western	11	18	43	60
Ma01	MWC06	15	1.611217	110.1638	Western	11	17	43	60
Ma14	MWC14	16	1.61125	110.1656	Western	11	17	41	59
Bt05	GG	17	1.30375	112.0739	Central	6	17	41	59
Bt04	BB	17	1.303917	112.074	Central	6	18	41	59
Bt03	BA68	17	1.304	112.0741	Central	6	18	42	52
Bt02	BA1001	17	2.169183	112.052	Central	6	18	43	60
Ka01	PO159	18	2.01595	112.9397	Central	11	18	41	59
Bt06	BA79	19	1.302	112.0766	Central	6	17	42	52
Bt07	BA113	19	1.301883	112.0768	Central	6	18	41	59
Ka03	PO161	20	2.168983	113.0517	Central	11	18	43	60
Ka05	PO158	21	2.16925	113.0521	Central	11	17	42	52
Ka06	PO160	21	2.169167	113.0521	Central	11	17	41	59
Bt08	BA38	22	1.308383	112.0803	Central	6	17	42	52
Ka04	PO162	23	2.169267	113.0521	Central	11	18	42	52
Bt01	BA69	24	1.301933	112.0767	Central	6	18	43	60
Bt02	BA119	24	1.303967	112.0741	Central	6	18	41	59
Br01	RZ13	25	3.72355	115.514	Northern	1	18	41	59
Br02	RZ14	25	3.724667	115.5142	Northern	1	18	43	60
Br03	RZ32	25	3.756667	115.4692	Northern	1	18	42	52
Br04	RZ34	25	3.7575	115.47	Northern	1	18	42	52
Mu03	RZ279	26	4.0505	114.8151	Northern	1	18	42	52
Mu04	RZ298	26	4.050583	114.815	Northern	1	18	41	59
Mu06	MHQ68	26	4.05065	114.8151	Northern	1	18	41	59
Seb01	SAB01	27	3.038533	113.9117	Central	14	18	43	60
Seb02	SAB13	27	3.038517	113.9118	Central	14	18	41	59
Eng02	EKL59	28	1.049817	111.6829	Central	11	17	43	60
Eng01	EKL58	29	1.049983	111.683	Central	11	17	42	52
Eng03	EKL61	30	1.050033	111.6831	Central	11	17	41	59
Mu07	MHQ62	31	4.04675	114.8352	Northern	1	18	43	60
Mu05	RZ299	31	4.037217	114.7458	Northern	1	18	43	60
Wil03	WILMAR14	31	3.501083	113.8251	Northern	14	18	43	60
Wil02	WILMAR12	31	3.484367	113.8315	Northern	14	18	42	52
Wil04	WILMAR16	31	3.501117	113.825	Northern	14	18	41	52
Mu02	MHQ69	32	4.136333	114.8945	Northern	1	18	43	60
Wil01	WILMAR11	33	3.484317	113.8314	Northern	14	18	43	60
Wil03	WILMAR17	34	4.136483	114.8947	Northern	14	18	43	60

*Note*:

* Zainudin *et al*. (2017) and Zulkefli and Zainudin (2022).

**Table 2:** Gene flow and genetic differentiation among populations of *L. leporinus*.

Locality	Distance (km)	Nucleotide subdivision (N_st_)^a^	Estimate of population subdivision (F_st_)^b^	Number of migrants per generation (N_m_)^b^	Genetic differentiation (Kst)^c^
Western-Central	328.043	0.74453	0.74005	0.09	0.35736*
Western-Northeast	587.874	0.84797	0.84182	0.04	0.42057*
Central-Northeast	288.702	0.42031	0.42027	0.34	0.18150*

*Notes*:

a Lynch and Crease (1990);

b Hudson, Slatkin, *et al*. (1992);

c Hudson, Boos, *et al*. (1992);

* significant at *p* < 0.05.

**Table 3:** DNA divergence of *L. leporinus* between localities.

Locality	Distance (km)	Nucleotide diversity (π)	Net nucleotide divergence (Da)	Haplotype diversity (Gst)
Western-Central	328.043	0.02840	0.05000	0.06821
Western-Northeast	587.874	0.04322	0.06667	0.09167
Central-Northeast	288.702	0.03134	0.01663	0.03380

**Table 4:** Shared haplotypes among *L. leporinus* in Sarawak populations.

Locality/clade	Haplotype ID	Frequency of shared haplotypes	Category total haplotypes per clade (1 hap = 1 unit)
Western	2	15 (2–3, 8–16, 18–20, 23)	25 unit
	11	8 (25–32)	
	12	2 (33–34)	
Central	13	2 (35–36)	10 unit
	15	2 (38–39)	
	16	4 (40, 42–44)	
	20	2 (47–48)	
Northeast	23	4 (52–55)	14 unit
	27	4 (59–62)	
	34	6 (69, 71, 73–76)	

## Data Availability

Supplementary Material.
