# Supplementary material for: Prioritising Conservation Area in Species Management Strategy for The Edible Bornean Giant River Frog Limnonectes leporinus Anderson 1923
Source: Trop Life Sci Res. 2024 Oct 7;35(3):57–75. doi: 10.21315/tlsr2024.35.3.3 (PMC11507967; doi:10.21315/tlsr2024.35.3.3)
Supplement: Supplementary file 1 [file TLSR_35-3-57-Suppl.pdf]

SUPPLEMENTARY MATERIAL

Prioritising Conservation Area in Species Management Strategy for The Edible Bornean Giant River Frog *Limnonectes leporinus* Anderson 1923

[1,2]Ramlah Zainudin,[*] [1]Elvy Quatrin Deka and [1]Julius Georgy

[1]Molecular Ecology Lab, Faculty of Resource Science and Technology, Universiti Malaysia Sarawak, 94300 Kota Samarahan, Sarawak, Malaysia
[2]Real Living Laboratory, Faculty of Resource Science and Technology, Universiti Malaysia Sarawak, 94300 Kota Samarahan, Sarawak, Malaysia

[*]Corresponding author: zramlah@unimas.my

Preparation of Data for Modelling *Limnonectes leporinus*

Species presence point is coordinate of every individual recorded using GPSmap 60CSx (GARMIN) during field samplings. The GPS reading was converted to decimal degree format saved in Excel using.csv format. The presence point Excel file will be generated in ArcGIS version 10.3 which only recognised information of the coordinates in decimal degree, requiring the X and Y be labelled as long_dd (longitude decimal degree) and lat_dd (latitude decimal degree). The formula to convert degree minute (DM) coordinates to decimal degree (DD) as follows: INT(A1)+(A1-INT(A1))/0.6

Species ID	Longitude	Latitude	Species	Lat_dd	Long_dd
Br01	03°43.413'	115°30.837'	Lleporinus	2.1301	113.0844
Br02	03°43.508'	115°30.107'	Lleporinus	2.13115	113.0842
Br03	03°45.400'	115°28.150'	Lleporinus	2.199083	113.0658
Br04	03°44.795'	115°28.899'	Lleporinus	1.049983	111.683
Mu01	04°03.030'	114°48.904'	Lleporinus	1.050033	111.6831
Mu02	04°03.045'	114°48.957'	Lleporinus	1.049967	111.6829
Mu03	04°03.076'	114°48.987'	Lleporinus	3.501117	113.825
Bt01	01°18.116'	112°04.604'	Lleporinus	3.484317	113.8314
Bt02	01°18.238'	112°04.444'	Lleporinus	3.48505	113.83
Bt03	01°18.155'	112°04.691'	Lleporinus	3.484633	113.8307
Bt04	01°14.358'	111°55.877'	Lleporinus	3.486517	113.8329
Bt05	01°14.357'	111°55.861'	Lleporinus	1.1612	110.2189
Bt06	01°18.178'	112°04.653'	Lleporinus	1.161217	110.2189
Bt07	01°18.281'	112°04.499'	Lleporinus	1.133717	110.2241
Bt08	01°18.161'	112°04.601'	Lleporinus	1.118367	110.211

Ka01	02°00.957'	112°56.381'	Lleporinus	1.141883	110.2165
Ka02	02°00.919'	112°56.375'	Lleporinus	1.715717	110.4459
Ka03	02°00.943'	112°56.369'	Lleporinus	1.714717	110.4438
Ka04	02°00.946'	112°56.362'	Lleporinus	4.136483	114.8947
Ka05	02°01.024'	112°56.245'	Lleporinus	4.1365	114.8956
Ka06	02°01.061'	112°56.459'	Lleporinus	4.060583	114.8717
Ma01	01°36.673'	110°09.828'	Lleporinus	4.037217	114.7458
Ma02	01°36.672'	110°09.819'	Lleporinus	4.04675	114.8352
Ma03	01°36.665'	110°09.835'	Lleporinus	1.744233	110.3187
Ma04	01°36.673'	110°09.925'	Lleporinus	1.302467	112.0764
Ma05	01°36.694'	110°09.754'	Lleporinus	1.308383	112.0803
Ma06	01°36.695'	110°09.755'	Lleporinus	1.607267	110.1943
Ma07	01°36.675'	110°09.888'	Lleporinus	3.72355	115.514
Ma08	01°36.660'	110°09.840'	Lleporinus	3.725133	115.5018
Ma09	01°36.679'	110°09.980'	Lleporinus	3.756667	115.4692
Ma10	01°36.698'	110°09.789'	Lleporinus	3.746583	115.4817
Ma11	01°36.701'	110°09.911'	Lleporinus	4.0505	114.8151
Ma12	01°36.498'	110°09.501'	Lleporinus	4.05075	114.816
Ma13	01°36.557'	110°09.609'	Lleporinus	4.051267	114.8165
Ma14	01°36.623'	110°09.758'	Lleporinus	1.301933	112.0767
Ma15	01°36.688'	110°09.803'	Lleporinus	1.303967	112.0741
Ma16	01°36.711'	110°09.903'	Lleporinus	1.302583	112.0782
Ba01	01°20.852'	110°02.352'	Lleporinus	1.2393	111.9313
Ba02	01°21.395'	110°01.992'	Lleporinus	1.239283	111.931
Ba03	01°30.118'	110°09.970'	Lleporinus	1.302967	112.0776
Ba04	01°32.530'	110°10.955'	Lleporinus	1.304683	112.075
Ba05	01°32.650'	110°10.885'	Lleporinus	1.302683	112.0767
Ba06	01°19.895'	110°06.012'	Lleporinus	2.01595	112.9397
Ba07	01°19.997'	110°06.135'	Lleporinus	2.015317	112.9396
Ku01	01°36.678'	110°11.861'	Lleporinus	2.015717	112.9395
Ku02	01°36.669'	110°11.829'	Lleporinus	2.015767	112.9394
Ku03	01°36.653'	110°11.834'	Lleporinus	2.017067	112.9374
Ku04	01°36.697'	110°11.853'	Lleporinus	2.017683	112.941

Ku05	01°36.703'	110°11.858'	Lleporinus	1.611217	110.1638
Ku06	01°36.247'	110°09.000'	Lleporinus	1.6112	110.1637
Ku07	01°36.733'	110°11.030'	Lleporinus	1.611083	110.1639
Ku08	01°36.755'	110°11.975'	Lleporinus	1.611217	110.1654
Ku09	01°36.711'	110°11.873'	Lleporinus	1.611567	110.1626
Pa01	01°10.906'	110°15.566'	Lleporinus	1.611583	110.1626
Pa02	01°10.929'	110°15.550'	Lleporinus	1.61125	110.1648
Pa03	01°12.289'	110°16.062'	Lleporinus	1.611	110.164
Pa04	01°10.937'	110°15.544'	Lleporinus	1.611317	110.1663
Pa05	01°10.887'	110°15.596'	Lleporinus	1.611633	110.1632
Pa06	01°10.884'	110°15.607'	Lleporinus	1.611683	110.1652
Ga01	01°41.495'	109°50.927'	Lleporinus	1.6083	110.1584
Ga02	01°41.501'	109°50.899'	Lleporinus	1.609283	110.1602
Seb01	03°02.312'	113°54.701'	Lleporinus	1.610383	110.1626
Seb02	03°02.311'	113°54.706'	Lleporinus	1.611467	110.1634
Ka07	02°07.806'	113°05.066'	Lleporinus	1.61185	110.1651
Ka08	02°07.869'	113°05.053'	Lleporinus	1.347533	110.0392
Ka09	02°11.945'	113°03.946'	Lleporinus	1.356583	110.0332
Eng01	01°02.999'	111°40.981'	Lleporinus	1.501967	110.1662
Eng02	01°03.002'	111°40.983'	Lleporinus	1.542167	110.1826
Eng03	01°02.998'	111°40.976'	Lleporinus	1.544167	110.1814
Wil01	03°30.067'	113°49.500'	Lleporinus	1.331583	110.1002
Wil02	03°29.059'	113°49.886'	Lleporinus	1.333283	110.1023
Wil03	03°29.103'	113°49.799'	Lleporinus	1.6113	110.1977
Wil04	03°29.078'	113°49.843'	Lleporinus	1.61115	110.1972
Wil05	03°29.191'	113°49.973'	Lleporinus	1.610883	110.1972
Pa07	01°09.672'	110°13.133'	Lleporinus	1.611617	110.1976
Pa08	01°09.673'	110°13.134'	Lleporinus	1.611717	110.1976
Pa09	01°08.023'	110°13.445'	Lleporinus	1.604117	110.15
Pa10	01°07.102'	110°12.661'	Lleporinus	1.612217	110.1838
Pa11	01°08.513'	110°12.989'	Lleporinus	1.612583	110.1996
KUHE57303	01°42.943'	110°26.752'	Lleporinus	1.61185	110.1979
KUHE57304	01°42.883'	110°26.629'	Lleporinus	1.181767	110.2594

Mu02	04°08.189'	114°53.679'	Lleporinus	1.18215	110.2592
Mu05	04°08.190'	114°53.734'	Lleporinus	1.204817	110.2677
Mu07	04°03.635'	114°52.301'	Lleporinus	1.182283	110.2591
Mu01	04°02.233'	114°44.746'	Lleporinus	1.18145	110.2599
Mu04	04°02.805'	114°50.109'	Lleporinus	1.1814	110.2601
Ga01	01°44.654'	110°19.122'	Lleporinus	1.691583	109.8488
Ga02	01°41.551'	110°50.867'	Lleporinus	1.691683	109.8483
Ga03	01°41.413'	110°50.935'	Lleporinus	3.038533	113.9117
Ga04	01°41.971'	109°50.348'	Lleporinus	3.038517	113.9118
Bt08	01°18.148'	112°04.581'	Lleporinus	3.49585	113.8317
Bt09	01°18.503'	112°04.818'	Lleporinus	3.495733	113.8315
Pa11	01°36.436'	110°11.657'	Lleporinus	3.495767	113.8317

There were seven environmental variables selected based on the species ecological requirement which were used as predictor variables in the suitable habitat modelling in which the model was then used as resistance map to run Circuitscape. The predictor variables were obtained from websites as following with the downloaded datasets in form of shapefile and .tiff formats:

Variables	Website	Dataset
1. Slopes	https://globalmaps.github.io/	asia_pac_gsr.tif
2. Intact forest landscape	http://www.intactforests.org/	ifl_2016.shp
3. Population density	https://sedac.ciesin.columbia.edu/data/set/gpw-v4-population-density-rev11	mys_pd_2020_1km_UNadj.tif
4. Water basins	https://datacatalog.worldbank.org/dataset/major-river-basins-world	major_basins_of_the world.shp
5. WorldClim temperature	https://www.worldclim.org/data/monthlywth.html	Wc2.1_2.5m_tmax_2019.tif
6. Elevation	https://datacatalog.worldbank.org/dataset/world-terrain-elevation-above-sea-level-ele-gis-data-global-solar-atlas	Wc2.1_2.5m-elev.tif
7. Land cover	https://www.gisresources.com/free-gis-data-land-cover-land-use-data/	Gm_lc>V1_1_2.tiff
8. Administrative boundary	https://www.diva-gis.org/gdata	MYS_adm2.shp

Prior to perform circuit theory using Circuitscape version 5.0, Julia interface was installed to allow the Circuitscape software generate data. The circuit analysis employed here was using haplotype data of DNA sequences which was generated in DNA Sequence Polymorphism (DNASP) version 6.12.01 (Rozas *et al.* 2017). The haplotype groups were identified as node ID in the species column and GPS reading were labelled as N and E instead of long_dd and lat_dd.

<table>
<tr><th>Haplotype data generated in DNASP</th><th>Prepared Excel file to compute in ArcGIS</th></tr>
<tr><td>
Hap_1: 1 [Ba07]
Hap_2: 15 [Ku05 Pa03 Ma04 Ma06 Ma03 Ba02 Ba05 Ba01 Ba06 Ku03 Ku02 Ku01 Ma09 Ba04 Ba03]
Hap_3: 1 [Pa04]
Hap_4: 1 [Pa05]
Hap_5: 1 [Ma01]
Hap_6: 1 Ma02]
Hap_7: 1 [Pa01]
Hap_8: 1 [Pa02]
Hap_9: 1 [Ma05]
Hap_10: 1 [Ku04]
Hap_11: 8 [Pa07 Pa08 Pa09 Ga02 Pa10 Pa11 Ku06 Ku07]
Hap_12: 2 [Ga01 Ga03]
Hap_13: 2 [Eng01 Eng03]
Hap_14: 1 [Eng02]
Hap_15: 2 [Seb01 Seb02]
Hap_16: 4 [Bt05 Bt04 Bt03 Ka02]
Hap_17: 1 [Ka01]
Hap_18: 1 [Bt06]
Hap_19: 1 [Ka03]
Hap_20: 2 [Ka05 Ka06]
Hap_21: 1 [Ka04]
Hap_22: 2 [Bt01 Bt02]
Hap_23: 4 [Br01 Br03 Br02 Br04]
Hap_24: 1 [Sa03]
Hap_25: 1 [Sa01]
Hap_26: 1 [Sa02]
Hap_27: 4 [Mu03 Mu04 Mu05 Mu06]
Hap_28: 1 [Mu01]
Hap_29: 1 [Mu02]
Hap_30: 1 [Bt09]
Hap_31: 1 [Bt08]
Hap_32: 1 [Ka09]
Hap_33: 1 [Ka07]
Hap_34: 6 [Ka08 Mulu05 Mulu07 Wil03 Wil02 Wil04]
Hap_35: 1 [Mulu02]
Hap_36: 1 [Wil01]
Hap_37: 1 [Mulu01]
</td><td>
<table>
<tr><th>Node ID</th><th>N</th><th>E</th></tr>
<tr><td>1</td><td>1.611917</td><td>110.1603</td></tr>
<tr><td>1</td><td>1.604117</td><td>110.1858</td></tr>
<tr><td>1</td><td>1.61115</td><td>110.1972</td></tr>
<tr><td>1</td><td>1.610883</td><td>110.1972</td></tr>
<tr><td>1</td><td>1.612067</td><td>110.1605</td></tr>
<tr><td>1</td><td>1.612</td><td>110.1604</td></tr>
<tr><td>1</td><td>1.611833</td><td>110.1602</td></tr>
<tr><td>1</td><td>1.612317</td><td>110.1608</td></tr>
<tr><td>1</td><td>1.367083</td><td>110.1688</td></tr>
<tr><td>1</td><td>1.3672</td><td>110.1691</td></tr>
<tr><td>1</td><td>1.36715</td><td>110.1689</td></tr>
<tr><td>1</td><td>1.383517</td><td>110.1688</td></tr>
<tr><td>1</td><td>1.61225</td><td>110.1607</td></tr>
<tr><td>1</td><td>1.367167</td><td>110.169</td></tr>
<tr><td>1</td><td>1.611083</td><td>110.1639</td></tr>
<tr><td>1</td><td>1.611217</td><td>110.1654</td></tr>
<tr><td>2</td><td>1.611483</td><td>110.1635</td></tr>
<tr><td>3</td><td>1.133717</td><td>110.2241</td></tr>
<tr><td>4</td><td>1.133833</td><td>110.2258</td></tr>
<tr><td>5</td><td>1.61215</td><td>110.1606</td></tr>
<tr><td>6</td><td>1.367317</td><td>110.1691</td></tr>
<tr><td>7</td><td>1.611567</td><td>110.1626</td></tr>
<tr><td>8</td><td>1.611617</td><td>110.1976</td></tr>
<tr><td>9</td><td>1.611</td><td>110.164</td></tr>
<tr><td>9</td><td>1.61165</td><td>110.1627</td></tr>
<tr><td>9</td><td>1.611717</td><td>110.1976</td></tr>
<tr><td>9</td><td>1.11825</td><td>110.2085</td></tr>
<tr><td>10</td><td>1.383683</td><td>110.1688</td></tr>
<tr><td>11</td><td>1.118367</td><td>110.211</td></tr>
<tr><td>12</td><td>1.133517</td><td>110.2227</td></tr>
<tr><td>13</td><td>1.133417</td><td>110.2227</td></tr>
<tr><td>14</td><td>1.6112</td><td>110.1637</td></tr>
<tr><td>15</td><td>1.611217</td><td>110.1638</td></tr>
<tr><td>16</td><td>1.61125</td><td>110.1656</td></tr>
</table>
</td></tr>
</table>

	17	1.30375	112.0739
	17	1.303917	112.074
	17	1.304	112.0741
	17	2.169183	112.052
	18	2.01595	112.9397
	19	1.302	112.0766
	19	1.301883	112.0768
	20	2.168983	113.0517
	21	2.16925	113.0521
	21	2.169167	113.0521
	22	1.308383	112.0803
	23	2.169267	113.0521
	24	1.301933	112.0767
	24	1.303967	112.0741
	25	3.72355	115.514
	25	3.724667	115.5142
	25	3.756667	115.4692
	25	3.7575	115.47
	26	4.0505	114.8151
	26	4.050583	114.815
	26	4.05065	114.8151
	27	3.038533	113.9117
	27	3.038517	113.9118
	28	1.049817	111.6829
	29	1.049983	111.683
	30	1.050033	111.6831
	31	2.16475	113.0856
	31	4.04675	114.8352
	31	4.037217	114.7458
	31	3.501083	113.8251
	31	3.484367	113.8315
	31	3.501117	113.825
	32	4.136333	114.8945
	33	3.484317	113.8314
	34	4.136483	114.8947

Dataset of DNA sequences used in this study:

>Ma01

GTCTACATTCTTATTCTACCTGGCTTCGGTATTATTTCACACGTTGTTGCTACTACTCCAGC
AAAAAAGAACCATTCGGCTATATGGGCATAGTCTGAGCCATATTATCAATTGGTCTCCTTGG
TTTTATTGTCTGAGCCCACCACATATTCACTACAGATCTAAACGTTGACACACGAGCATATT
TTACTTCAGCCACAATAATCATTGCTATTCCCACTGGGGTTAAAGTTTTTAGTTGGCTGGCC
ACCATGCACGGCGGTGTTATTAAATGAGAAGCCCCCATATTGTGAGCACTAGGCTTTATTT
TCTTATTCACCATCGGAGGCCTAACCGGCATTGTTCTTGCCAACTCCTCAATCGATATTGTT
CTTCACGACACCTACTACGTTGTCGCCCATTTCCACTATGTATTATCAATAGGAGCAGTCTT
TGCTATCATAGCCGGATTTGTTCACTGATTCCC

>Ma05

GTCTACATTCTTATTCTACCTGGCTTCGGTATTATTTCACACGTTGTAGCATACTACTCCAG
CAAAAAAGAACCATTCGGCTATATGGGCATAGTCTGAGCCATATTATCAATTGGTCTCGTC
GCTTTTATTGTCTGAGCCCACCCAATATTCACTACAGATCTAAACGTTGACACACGAGCATA
TTTTACTTCAGCCACAATAATCATTGCTATTCCCACTGGTGTTAAAGTTTTTAGTTGGCTGG
CCACCATGCACGGCGGTGTTATTAAATGAGAAGCTCCAATATTGTGAGCATTAGGCTTCAT
TTTCTTATTCACCATCGGAGGCCTAACCGGCATTGTTCTCGCCAACTCCTCAATCGATATTG
TTCTTCACGACACCTACTACGTAGTCGCCCATTTCCACTATGTATTATCAATAGGAGCCGTC
TTTGCTATCATAGCCGGATTTGTTCACTGATTCCC

>Ma02

GTCTACATTCTTATTCTACCTGGCTTCGGTATTATTTCACACGTTGTAGCATACTACTCCAG
CAAAAAAGAACCATTCGGCTATATGGGCATAGTCTGAGCCATATTATCAATTGGTCTCCTTG
GTTTTATTGTCTGAGCCCACCACATATTCACTACAGATCTAAACGTTGACACACGAGCATAT
TTTACTTCAGCCACAATAATCATTGCTATTCCCACTGGTGTTAAAGTTTTTAGTTGGCTGGC
CACCATGCACGGCGGTGTTATTAAATGAGAAGCTCCAATATTGTGAGCATTAGGCTTCATTT
TCTTATTCACCATCGGAAGCCTAACCGGCATTGTTCTCGCCAACTCCTCAATCGATATTGTT
CTTCACGACACCTACTACGTAGTCGCCCATTTCCACTATGTATTATCAATAGGAGCAGTCTT
TGCTATCATAGCCGGATTTGTTCACTGATTCCC

>Pa01

GTCTACATTCTTATTCTACCTGGCTTCGGTATTATTTCACACGTTGTAGCATACTACTCCAG
CAAAAAAGAACCATTCGGCTATATGGGCATAGTCTGAGCCATATTATCAATTGGTCTCCTTG
GTTTTATTGTCTGAGCCCACCACATATTCACTACAGATCTAAACGTTGACACACGAGCATAT
TTTACTTCAGCCACAATAATCATTGCTATTCCCACTGGTGTTAAAGTTTTTAGTTGGCTGGC
CACCATGCACGGCGGTGTTATTAAATGAGAAGCTCCAATATTGTGAGCATTAGGCTTCATTT
TCTTATTCACCATCGGAGGCCTAACCGGCATTGTTCTCGCCAACTCCTCAATCGATATTGTT
CTTCACGACACCTACTACGTAGTCGCCCATTTCCACTATGTTTTATCAATAGGAGCCGTCTT
TGCTATCATAACCGGATTTGTTCACTGATTCCC

>Ma04

GTCTACATTCTTATTCTACCTGGCTTCGGTATTATTTCACACGTTGTAGCATACTACTCCAG
CAAAAAAGAACCATTCGGCTATATGGGCATAGTCTGAGCCATATTATCAATTGGTCTCCTTG
GTTTTATTGTCTGAGCCCACCACATATTCACTACAGATCTAAACGTTGACACACGAGCATAT
TTTACTTCAGCCACAATAATCATTGCTATTCCCACTGGTGTTAAAGTTTTTAGTTGGCTGGC
CACCATGCACGGCGGTGTTATTAAATGAGAAGCTCCAATATTGTGAGCATTAGGCTTCATTT
TCTTATTCACCATCGGAGGCCTAACCGGCATTGTTCTCGCCAACTCCTCAATCGATATTGTT
CTTCACGACACCTACTACGTAGTCGCCCATTTCCACTATGTATTATCAATAGGAGCCGTCTT
TGCTATCATAGCCGGATTTGTTCACTGATTCCC

>Ma03

GTCTACATTCTTATTCTACCTGGCTTCGGTATTATTTCACACGTTGTAGCATACTACTCCAG
CAAAAAAGAACCATTCGGCTATATGGGCATAGTCTGAGCCATATTATCAATTGGTCTCCTTG
GTTTTATTGTCTGAGCCCACCACATATTCACTACAGATCTAAACGTTGACACACGAGCATAT
TTTACTTCAGCCACAATAATCATTGCTATTCCCACTGGTGTTAAAGTTTTTAGTTGGCTGGC
CACCATGCACGGCGGTGTTATTAAATGAGAAGCTCCAATATTGTGAGCATTAGGCTTCATTT
TCTTATTCACCATCGGAGGCCTAACCGGCATTGTTCTCGCCAACTCCTCAATCGATATTGTT
CTTCACGACACCTACTACGTAGTCGCCCATTTCCACTATGTATTATCAATAGGAGCCGTCTT
TGCTATCATAGCCGGATTTGTTCACTGATTCCC

>Pa04

GTCTACATTCTTATTCTACCTGGCTTCGGTATTATTTCACACGTTGTAGCATACTACTCCAG
CAAAAAAGAACCATTCGGCTATATGGGCATAGTCTGAGCCATATTATCAATTGGTCTCCTTG
GTTTTATTGTCTGAGCCCACCACATATTCACTACAGATCTAAACGTTGACACACGAGCATAT
TTTACTTCAGCCACAATAATCATTGCTATTCCCACTGGTGTTAAAGTTTTTAGTTGGCTGGC
CACCATGCACGGCGGTGTTATCAAATGAGAAGCTCCAATATTGTGAGCATTAGGCTTCATT
TTCTTATTCACCATCGGAGGCCTAACCGGCATTGTTCTCGCCAACTCCTCAATCGATATTGT
TCTTCACGACACCTACTACGTAGTCGCCCATTTCCACTATGTAATTACTATAGGAGCCGTCT
TTGCTATCATAGCCGGATTTGTTCACTGATTCCC

>Pa05

GTCTACATTCTTATTCTACCTGGCTTCGGAATTATTTCACACGTTGTAGCATACTACTCCAG
CAAAAAAGAACCATTCGGCTATATGGGCATAGTCTGAGCCATATTATCAATTGGTCTTCTTG
GTTTTATTGTCTGAGCCCACCACATATTCACTACAGATCTAAACGTTGACACACGAGCATAT
TTTACTTCCGCCACAATAATCATTGCTATTCCCACTGGTGTTAAAGTTTTTAGTTGGCTGGC
CACCATGCACGGCGGTGTTATCAAATGAGAAGCTCCAATATTGTGAGCATTAGGCTTCATT
TTCTTATTCACCATCGGAGGCCTAACCGGCATTGTTCTCGCCAACTCCTCAATCGATATTGT
TCTTCACGACACCTACTACGTAGTCGCCCATTTCCACTATGTATTATCAATAGGAGCCGTCT
TTGCTATCATAGCCGGATTTGTTCACTGATTCCC

>Pa02

GTCTACATTCTTATTCTACCTGGCTTCGGTATTATTTCACACGTTGTAGCATACTACTCCAG
CAAAAAAGAACCATTCGGCTATATGGGCATAGTTTGAGCCATATTATCAATTGGTCTCCTTG

GTTTTATTGTCTGAGCCCACCACATATTCACTACAGATCTAAACGTTGACACACGAGCATAT
TTTACTTCAGCCACAATAATCATTGCTATTCCCACTGGTGTTAAAGTTTTTAGTTGGCTGGC
CACCATGCACGGCGGTGTTATTAAATGAGAAGCTCCAATATTGTGAGCATTAGGCTTCATTT
TCTTATTCACCATCGGAGGCCTAACCGGCATTGTTCTCGCCAACTCCTCAATCGATATTGTT
CTTCACGACACCTACTACGTAGTCGCCCATTTCCACTATGTATTATCAATAGGAGCCGTCTT
TGCTATCATAGCCGGATTTGTTCACTGATTCCC

>Bt05

GTCTACATTCTTATTCTACCTGGCTTCGGTATTATCTCACACGTTGTAGCATACTACTCCAG
CAAAAAAGAATCATTCGGCTACATAGGTATAGTCTGAGCTATATTATCAATTGGCCTTCTTG
GTTTCATTGTTTGAGCCCACCATATATTCACCACAGACCTAAACGTTGACACACGAGCATAT
TTTACTTCAGCCACAATAATCATTGCTATTCCCACTGGCGTTAAAGTTTTCAGTTGATTGGC
TACCATGCACGGTGGCATCATTAAATGAGAAGCTCCAATACTATGAGCATTAGGCTTCATCT
TCTTATTTACCATCGGAGGCCTAACCGGCATTGTTCTTGCCAACTCCTCTATCGATATTGTT
CTTCACGATACCTACTACGTAGTTGCTCACTTCCACTACGTATTATCAATAGGAGCCGTCTT
TGCTATCATAGCCGGATTTGTCCACTGATTCCC

>Bt04

GTCTACATTCTTATTCTACCTGGCTTCGGTATTATCTCACACGTTGTAGCATACTACTCCAG
CAAAAAAGAATCATTCGGCTACATAGGTATAGTCTGAGCTATATTATCAATTGGCCTTCTTG
GTTTCATTGTTTGAGCCCACCATATATTCACCACAGACCTAAACGTTGACACACGAGCATAT
TTTACTTCAGCCACAATAATCATTGCTATTCCCACTGGCGTTAAAGTTTTCAGTTGATTGGC
TACCATGCACGGTGGCATCATTAAATGAGAAGCTCCAATACTATGAGCATTAGGCTTCATCT
TCTTATTTACCATCGGAGGCCTAACCGGCATTGTTCTTGCCAACTCCTCTATCGATATTGTT
CTTCACGATACCTACTACGTAGTTGCTCACTTCCACTACGTATTATCAATAGGAGCCGTCTT
TGCTATCATAGCCGGATTTGTCCACTGATTCCC

>Ba02

GTCTACATTCTTATTCTACCTGGCTTCGGTATTATTTCACACGTTGTAGCATACTACTCCAG
CAAAAAAGAACCATTCGGCTATATGGGCATAGTCTGAGCCATATTATCAATTGGTCTCCTTG
GTTTTATTGTCTGAGCCCACCACATATTCACTACAGATCTAAACGTTGACACACGAGCATAT
TTTACTTCAGCCACAATAATCATTGCTATTCCCACTGGTGTTAAAGTTTTTAGTTGGCTGGC
CACCATGCACGGCGGTGTTATTAAATGAGAAGCTCCAATATTGTGAGCATTAGGCTTCATTT
TCTTATTCACCATCGGAGGCCTAACCGGCATTGTTCTCGCCAACTCCTCAATCGATATTGTT
CTTCACGACACCTACTACGTAGTCGCCCATTTCCACTATGTATTATCAATAGGAGCCGTCTT
TGCTATCATAGCCGGATTTGTTCACTGATTCCC

>Ba05

GTCTACATTCTTATTCTACCTGGCTTCGGTATTATTTCACACGTTGTAGCATACTACTCCAG
CAAAAAAGAACCATTCGGCTATATGGGCATAGTCTGAGCCATATTATCAATTGGTCTCCTTG
GTTTTATTGTCTGAGCCCACCACATATTCACTACAGATCTAAACGTTGACACACGAGCATAT
TTTACTTCAGCCACAATAATCATTGCTATTCCCACTGGTGTTAAAGTTTTTAGTTGGCTGGC
CACCATGCACGGCGGTGTTATTAAATGAGAAGCTCCAATATTGTGAGCATTAGGCTTCATTT

TCTTATTCACCATCGGAGGCCTAACCGGCATTGTTCTCGCCAACTCCTCAATCGATATTGTT
CTTCACGACACCTACTACGTAGTCGCCCATTTCCACTATGTATTATCAATAGGAGCCGTCTT
TGCTATCATAGCCGGATTTGTTCACTGATTCCC

>Ba03

GTCTACATTCTTATTCTACCTGGCTTCGGTATTATTTCACACGTTGTAGCATACTACTCCAG
CAAAAAAGAACCATTCGGCTATATGGGCATAGTCTGAGCCATATTATCAATTGGTCTCCTTG
GTTTTATTGTCTGAGCCCACCACATATTCACTACAGATCTAAACGTTGACACACGAGCATAT
TTTACTTCAGCCACAATAATCATTGCTATTCCCACTGGTGTTAAAGTTTTTAGTTGGCTGGC
CACCATGCACGGCGGTGTTATTAAATGAGAAGCTCCAATATTGTGAGCATTAGGCTTCATTT
TCTTATTCACCATCGGAGGCCTAACCGGCATTGTTCTCGCCAACTCCTCGATCGATATTGTT
CTTCACGACACCTACTACGTAGTCGCCCATTTCCACTATGTATTATCAATAGGAGCCGTCTT
TGCTATCATAGCCGGATTTGTTCACTGATTCCC

>Ba01

GTCTACATTCTTATTCTACCTGGCTTCGGTATTATTTCACACGTTGTAGCATACTACTCCAG
CAAAAAAGAACCATTCGGCTATATGGGCATAGTCTGAGCCATATTATCAATTGGTCTCCTTG
GTTTTATTGTCTGAGCCCACCACATATTCACTACAGATCTAAACGTTGACACACGAGCATAT
TTTACTTCAGCCACAATAATCATTGCTATTCCCACTGGTGTTAAAGTTTTTAGTTGGCTGGC
CACCATGCACGGCGGTGTTATTAAATGAGAAGCTCCAATATTGTGAGCATTAGGCTTCATTT
TCTTATTCACCATCGGAGGCCTAACCGGCATTGTTCTCGCCAACTCCTCAATCGATATTGTT
CTTCACGACACCTACTACGTAGTCGCCCATTTCCACTATGTATTATCAATAGGAGCCGTCTT
TGCTATCATAGCCGGATTTGTTCACTGATTCCC

>Ba07

GTCTACATTCTTATTCTACCTGGCTTCGGTATTATTTCACACGTTGTAGCATACTACTCCAG
CAAAAAAGAACCATTCGGCTATATGGGCATAGTCTGAGCCATATTATCAATTGGTCTCCTTG
GTTTTATTGTCTGAGCCCACCACATATTCACTACAGATCTAAACGTTGACACACGAGCATAT
TTTACTTCAGCCACAATAATCATTGCTATTCCCACTGGTGTTAAAGTTTTTAGTTGGCTGGC
CACCATGCACGGCGGTGTTATCAAATGAGAAGCTCCAATATTGTGAGCATTAGGCTTCATT
TTCTTATTCACCATCGGAGGCCTAACCGGCATTGTTCTCGCCAACTCCTCAATCGATATTGT
TCTTCACGACACCTACTACGTAGTCGCCCATTTCCACTATGTATTATCAATAGGAGCCGTTT
TTGCTATCATAGCCGGATTTGTTCACTGATTCCC

>Ba04

GTCTACATTCTTATTCTACCTGGCTTCGGTATTATTTCACACGTTGTAGCATACTACTCCAG
CAAAAAAGAACCATTCGGCTATATGGGCATAGTCTGAGCCATATTATCAATTGGTCTCCTTG
GTTTTATTGTCTGAGCCCACCACATATTCACTACAGATCTAAACGTTGACACACGAGCATAT
TTTACTTCAGCCACAATAATCATTGCTATTCCCACTGGTGTTAAAGTTTTTAGTTGGCTGGC
CACCATGCACGGCGGTGTTATTAAATGAGAAGCTCCAATATTGTGAGCATTAGGCTTCATTT
TCTTATTCACCATCGGAGGCCTAACCGGCATTGTTCTCGCCAACTCCTCAATCGATATTGTT
CTTCACGACACCTACTACGTAGTCGCCCATTTCCACTATGTATTATCAATAGGAGCCGTCTT
TGCTATCATAGCCGGATTTGTTCACTGATTCCC

>Ba06

GTCTACATTCTTATTCTACCTGGCTTCGGTATTATTTCACACGTTGTAGCATACTACTCCAG
CAAAAAAGAACCATTCGGCTATATGGGCATAGTCTGAGCCATATTATCAATTGGTCTCCTTG
GTTTTATTGTCTGAGCCCACCACATATTCACTACAGATCTAAACGTTGACACACGAGCATAT
TTTACTTCAGCCACAATAATCATTGCTATTCCCACTGGTGTTAAAGTTTTTAGTTGGCTGGC
CACCATGCACGGCGGTGTTATTAAATGAGAAGCTCCAATATTGTGAGCATTAGGCTTCATTT
TCTTATTCACCATCGGAGGCCTAACCGGCATTGTTCTCGCCAACTCCTCAATCGATATTGTT
CTTCACGACACCTACTACGTAGTCGCCCATTTCCACTATGTATTATCAATAGGAGCCGTCTT
TGCTATCATAGCCGGATTTGTTCACTGATTCCC

>Br01

GTCTACATTCTTATTCTACCTGGCTTCGGTATTATCTCACACGTTGTAGCATACTACTCCAG
CAAAAAAGAATCATTCGGCTATATAGGTATAGTCTGAGCTATATTATCAATTGGTCTTCTTG
GTTTCATTGTTTGAGCTCACCACATATTCACCACAGACCTAAACGTTGACACACGAGCATAT
TTTACTTCAGCCACAATAATCATTGCTATTCCCACTGGCGTTAAAGTTTTCAGTTGACTGGC
TACCATGCACGGCGGTATCATTAAATGAGAAGCTCCAATACTATGAGCATTAGGCTTCATCT
TCTTATTTACCATCGGAGGCCTAACCGGCATTGTTCTTGCCAACTCCTCTATCGATATTGTT
CTTCATGATACCTACTACGTAGTTGCTCACTTCCACTACGTACTATCAATAGGAGCCGTCTT
TGCTATCATAGCTGGATTCGTCCACTGATTCCC

>Br02

GTCTACATTCTTATTCTACCTGGCTTCGGTATTATCTCACACGTTGTAGCATACTACTCCAG
CAAAAAAGAATCATTCGGCTATATAGGTATAGTCTGAGCTATATTATCAATTGGTCTTCTTG
GTTTCATTGTTTGAGCTCACCACATATTCACCACAGACCTAAACGTTGACACACGAGCATAT
TTTACTTCAGCCACAATAATCATTGCTATTCCCACTGGCGTTAAAGTTTTCAGTTGACTGGC
TACCATGCACGGCGGTATCATTAAATGAGAAGCTCCAATACTATGAGCATTAGGCTTCATCT
TCTTATTTACCATCGGAGGCCTAACCGGCATTGTTCTTGCCAACTCCTCTATCGATATTGTT
CTTCATGATACCTACTACGTAGTTGCTCACTTCCACTACGTACTATCAATAGGAGCCGTCTT
TGCTATCATAGCTGGATTCGTCCACTGATTCCC

>Br03

GTCTACATTCTTATTCTACCTGGCTTCGGTATTATCTCACACGTTGTAGCATACTACTCCAG
CAAAAAAGAATCATTCGGCTATATAGGTATAGTCTGAGCTATATTATCAATTGGTCTTCTTG
GTTTCATTGTTTGAGCTCACCACATATTCACCACAGACCTAAACGTTGACACACGAGCATAT
TTTACTTCAGCCACAATAATCATTGCTATTCCCACTGGCGTTAAAGTTTTCAGTTGACTGGC
TACCATGCACGGCGGTATCATTAAATGAGAAGTCCAATACTATGAGCATTAGGCTTCATCTT
CTTATTTACCATCGGAGGCCTAACCGGCATTGTTCTTGCCAACTCCTCTATCGATATTGTTC
TTCATGATACCTACTACGTAGTTGCTCACTTCCACTACGTACTATCAATAGGAGCCGTCTTT
GCTATCATAGCTGGATTCGTCCACTGATTCCC

>Br04

GTCTACATTCTTATTCTACCTGGCTTCGGTATTATCTCACACGTTGTAGCATACTACTCCAG
CAAAAAAGAATCATTCGGCTATATAGGTATAGTCTGAGCTATATTATCAATTGGTCTTCTTG
GTTTCATTGTTTGAGCTCACCACATATTCACCACAGACCTAAACGTTGACACACGAGCATAT
TTTACTTCAGCCACAATAATCATTGCTATTCCCACTGGCGTTAAAGTTTTCAGTTGACTGGC
TACCATGCACGGCGGTATCATTAAATGAGAAGCTCCAATACTATGAGCATTAGGCTTCATCT
TCTTATTTACCATCGGAGGCCTAACCGGCATTGTTCTTGCCAACTCCTCATCGATATTGTTC
TTCATGATACCTACTACGTAGTTGCTCACTTCCACTACGTACTATCAATAGGAGCCGTCTTT
GCTATCATAGCTGGATTCGTCCACTGATTCCC

>Ma06

GTCTACATTCTTATTCTACCTGGCTTCGGTATTATTTCACACGTTGTAGCATACTACTCCAG
CAAAAAAGAACCATTCGGCTATATGGGCATAGTCTGAGCCATATTATCAATTGGTCCCTTG
GTTTTATTGTCTGAGCCCACCACATATTCACTACAGATCTAAACGTTGACACACGAGCATAT
TTTACTTCAGCCCAATAATCATTGCTATTCCCACTGGTGTTAAAGTTTTTAGTTGGCTGGCC
ACCATGCACGGCGGTGTTATTAAATGAGAAGCTCCAATATTGTGAGCATTAGGCTTCATTTT
CTTATTCACCATCGGAGGCCTAACCGGCATTGTTCTCGCCAACTCCTCAATCGATATTGTTC
TTCACGACACCTACTACGTAGTCGCCCATTTCCACTATGTATTATCAATAGGAGCCGTCTTT
GCTATCATAGCCGGATTTGTTCACTGATTCCC

>Ma09

GTCTACATTCTTATTCTACCTGGCTTCGGTATTATTTCACACGTTGTAGCATACTACTCCAG
CAAAAAAGAACCATTCGGCTATATGGGCATAGTCTGAGCCATATTATCAATTGGTCTCCTTG
GTTTTATTGTCTGAGCCCACCACATATTCACTACAGATCTAAACGTTGACACACGAGCATAT
TTTACTTCAGCCACAATAATCATTGCTATTCCCACTGGTGTTAAAGTTTTTAGTTGGCTGGC
CACCATGCACGGCGGTGTTATTAAATGAGAAGCTCCAATATTGTGAGCATTAGGCTTCATTT
TCTTATTCACCATCGGAGGCCTAACCGGCATTGTTCTCGCCAACTCCTCAATCGATATTGTT
CTTCACGACACCTACTACGTAGTCGCCCATTTCCACTATGTATTATCAATAGGAGCCGTCTT
TGCTATCATAGCCGGATTTGTTCACTGATTCCC

>Ku03

GTCTACATTCTTATTCTACCTGGCTTCGGTATTATTTCACACGTTGTAGCATACTACTCCAG
CAAAAAAGAACCATTCGGCTATATGGGCATAGTCTGAGCCATATTATCAATTGGTCTCCTTG
GTTTTATTGTCTGAGCCCACCACATATTCACTACAGATCTAAACGTTGACACACGAGCATAT
TTTACTTCAGCCACAATAATCATTGCTATTCCCACTGGTGTTAAAGTTTTTAGTTGGCTGGC
CACCATGCACGGCGGTGTTATTAAATGAGAAGCTCCAATATTGTGAGCATTAGGCTTCATTT
TCTTATTCACCATCGGAGGCCTAACCGGCATTGTTCTCGCCAACTCCTCAATCGATATTGTT
CTTCACGACACCTACTACGTAGTCGCCCATTTCCACTATGTATTATCAATAGGAGCCGTCTT
TGCTATCATAGCCGGATTTGTTCACTGATTCCC

>Ku05

GTCTACATTCTTATTCTACCTGGCTTCGGTATTATTTCACACGTTGTAGCATACTACTCCAG
CAAAAAAGAACCATTCGGCTATATGGGCATAGTCTGAGCCATATTATCAATTGGTCTCCTTG

GTTTTATTGTCTGAGCCCACCACATATTCACTACAGATCTAAACGTTGACACACGAGCATAT
TTTACTTCAGCCACAATAATCATTGCTATTCCCACTGGTGTTAAAGTTTTTAGTTGGCTGGC
CACCATGCACGGCGGTGTTATCAAATGAGAAGCTCCAATATTGTGAGCATTAGGCTTCATT
TTCTTATTCACCATCGGAGGCCTAACCGGCATTGTTCTCGCCAACTCCTCAATCGATATTGT
TCTTCACGACACCTACTACGTAGTCGCCCATTTCCACTATGTATTATCAATAGGAGCCGTCT
TTGCTATCATAGCCGGATTTGTTCACTGATTCCC

>Ku01

GTCTACATTCTTATTCTACCTGGCTTCGGTATTATTTCACACGTTGTAGCATACTACTCCAG
CAAAAAAGAACCATTCGGCTATATGGGCATAGTCTGAGCCATATTATCAATTGGTCTCCTTG
GTTTTATTGTCTGAGCCCACCACATATTCACTACAGATCTAAACGTTGACACACGAGCATAT
TTTACTTCAGCCACAATAATCATTGCTATTCCCACTGGTGTTAAAGTTTTTAGTTGGCTGGC
CACCATGCACGGCGGTGTTATTAAATGAGAAGCTCCAATATTGTGAGCATTAGGCTTCATTT
TCTTATTCACCATCGGAGGCCTAACCGGCATTGTTCTCGCCAACTCCTCAATCGATATTGTT
CTTCACGACACCTACTACGTAGTCGCCCATTTCCACTATGTATTATCAATAGGAGCCGTCTT
TGCTATCATAGCCGGATTTGTTCACTGATTCCC

>Ku02

GTCTACATTCTTATTCTACCTGGCTTCGGTATTATTTCACACGTTGTAGCATACTACTCCAG
CAAAAAAGAACCATTCGGCTATATGGGCATAGTCTGAGCCATATTATCAATTGGTCTCCTTG
GTTTTATTGTCTGAGCCCACCACATATTCACTACAGATCTAAACGTTGACACACGAGCATAT
TTTACTTCAGCCACAATAATCATTGCTATTCCCACTGGTGTTAAAGTTTTTAGTTGGCTGGC
CACCATGCACGGCGGTGTTATTAAATGAGAAGCTCCAATATTGTGAGCATTAGGCTTCATTT
TCTTATTCACCATCGGAGGCCTAACCGGCATTGTTCTCGCCAACTCCTCAATCGATATTGTT
CTTCACGACACCTACTACGTAGTCGCCCATTTCCACTATGTATTATCAATAGGAGCCGTCTT
TGCTATCATAGCCGGATTTGTTCACTGATTCCC

>Mu03

GTCTACATTCTTATTCTACCTGGCTTCGGTATTATCTCACACGTTGTAGCATACTACTCCAG
CAAAAAAGAATCATTCGGCTATATGGGCATAGTCTGAGCTATATTATCAATTGGCCTTCTTG
GTTTTATTGTTTGAGCTCACCATATATTCACCACAGACCTAAACGTTGACACACGAGCATAC
TTTACTTCAGCCACAATAATCATTGCTATTCCCACTGGCGTTAAAGTTTTCAGTTGACTGGC
TACCATGCACGGTGGTATCATTAAATGAGAAGCTCCAATACTATGAGCATTAGGCTTCATCT
TCTTATTTACCATCGGAGGCCTAACCGGCATTGTTCTTGCCAACTCCTCTATCGATATTGTT
CTTCATGACACCTACTACGTGGTTGCTCACTTCCACTACGTACTATCAATAGGAGCCGTCTT
TGCTATCATAGCTGGATTCGTCCACTGATTCCC

>Mu04

GTCTACATTCTTATTCTACCTGGCTTCGGTATTATCTCACACGTTGTAGCATACTACTCCAG
CAAAAAAGAATCATTCGGCTATATGGGCATAGTCTGAGCTATATTATCAATTGGCCTTCTTG
GTTTTATTGTTTGAGCTCACCATATATTCACCACAGACCTAAACGTTGACACACGAGCATAC
TTTACTTCAGCCACAATAATCATTGCTATTCCCACTGGCGTTAAAGTTTTCAGTTGACTGGC
TACCATGCACGGTGGTATCATTAAATGAGAAGCTCCAATACTATGAGCATTAGGCTTCATCT

TCTTATTTACCATCGGAGGCCTAACCGGCATTGTTCTTGCCAACTCCTCTATCGATATTGTT
CTTCATGACACCTACTACGTGGTTGCTCACTTCCACTACGTACTATCAATAGGAGCCGTCTT
TGCTATCATAGCTGGATTCGTCCACTGATTCCC

>Mu05

GTCTACATTCTTATTCTACCTGGCTTCGGTATTATCTCACACGTTGTAGCATACTACTCCAG
CAAAAAAGAATCATTCGGCTATATGGGCATAGTCTGAGCTATATTATCAATTGGCCTTCTTG
GTTTTATTGTTTGAGCTCACCATATATTCACCACAGACCTAAACGTTGACACACGAGCATAC
TTTACTTCAGCCACAATAATCATTGCTATTCCCACTGGCGTTAAAGTTTTCAGTTGACTGGC
TACCATGCACGGTGGTATCATTAAATGAGAAGCTCCAATACTATGAGCATTAGGCTTCATCT
TCTTATTTACCATCGGAGGCCTAACCGGCATTGTTCTTGCCAACTCCTCTATCGATATTGTT
CTTCATGACACCTACTACGTGGTTGCTCACTTCCACTACGTACTATCAATAGGAGCCGTCTT
TGCTATCATAGCTGGATTCGTCCACTGATTCCC

>Pa03

GTCTACATTCTTATTCTACCTGGCTTCGGTATTATTTCACACGTTGTAGCATACTACTCCAG
CAAAAAAGAACCATTCGGCTATATGGGCATAGTCTGAGCCATATTATCAATTGGTCTCCTTG
GTTTTATTGTCTGAGCCCACCACATATTCACTACAGATCTAAACGTTGACACACGAGCATAT
TTTACTTCAGCCACAATAATCATTGCTATTCCCACTGGTGTTAAAGTTTTTAGTTGGCTGGC
CACCATGCACGGCGGTGTTATCAAATGAGAAGCTCCAATATTGTGAGCATTAGGCTTCATT
TTCTTATTCACCATCGGAGGCCTAACCGGCATTGTTCTCGCCAACTCCTCAATCGATATTGT
TCTTCACGACACCTACTACGTAGTCGCCCATTTCCACTATGTATTATCAATAGGAGCCGTCT
TTGCTATCATAGCCGGATTTGTTCACTGATTCCC

>Bt03

GTCTACATTCTTATTCTACCTGGCTTCGGTATTATCTCACACGTTGTAGCATACTACTCCAG
CAAAAAAGAATCATTCGGCTACATAGGTATAGTCTGAGCTATATTATCAATTGGCCTTCTTG
GTTTCATTGTTTGAGCCCACCATATATTCACCACAGACCTAAACGTTGACACACGAGCATAT
TTTACTTCAGCCACAATAATCATTGCTATTCCCACTGGCGTTAAAGTTTTCAGTTGATTGGC
TACCATGCACGGTGGCATCATTAAATGAGAAGCTCCAATACTATGAGCATTAGGCTTCATCT
TCTTATTTACCATCGGAGGCCTAACCGGCATTGTTCTTGCCAACTCCTCTATCGATATTGTT
CTTCACGATACCTACTACGTAGTTGCTCACTTCCACTACGTATTATCAATAGGAGCCGTCTT
TGCTATCATAGCCGGATTTGTCCACTGATTCCC

>Bt01

GTCTACATTCTTATTCTACCTGGCTTCGGTATTATCTCACACGTTGTAGCATACTACTCCAG
CAAAAAAGAATCATTCGGCTACATAGGTATAGTCTGAGCTATATTATCAATTGGTCTTCTTG
GTTTCATTGTTTGAGCCCACCATATATTCACTACAGACCTAAACGTTGACACACGAGCATAT
TTTACTTCAGCCACAATAATCATTGCTATTCCCACTGGCGTTAAAGTTTTCAGTTGATTGGC
TACCATGCACGGTGGCATCATTAAATGAGAAGCTCCAATACTATGAGCATTAGGCTTCATCT
TCTTATTTACCATCGGAGGCCTAACCGGCATTGTTCTTGCCAACTCCTCTATCGATATTGTT
CTTCACGATACCTACTACGTAGTTGCTCACTTCCACTACGTATTATCAATAGGAGCCGTCTT
TGCTATCATAGCCGGATTTGTCCACTGATTCCC

>Bt06

GTCTACATTCTTATTCTACCTGGCTTCGGTATTATCTCACACGTTGTAGCATACTACTCCAG
CAAAAAAGAATCATTCGGCTACATAGGTATAGTCTGAGCTATATTATCAATTGGTCTTCTTG
GTTTCATTGTTTGAGCCCACCATATATTCACCACAGACCTAAACGTTGACACACGAGCATAT
TTTACTTCAGCCACAATAATTATTGCTATTCCCACTGGCGTTAAAGTTTTCAGTTGATTGGCT
ACCATGCACGGTGGCATCATTAAATGAGAAGCTCCAATACTATGAGCATTAGGCTTCATCTT
CTTATTTACCATCGGAGGCCTAACCGGCATTGTTCTTGCCAACTCCTCTATCGATATTGTTC
TTCACGATACCTACTACGTAGTTGCTCACTTCCACTACGTATTATCAATAGGAGCCGTCTTT
GCTATCATAGCCGGATTTGTCCACTGATTCCC

>Bt02

GTCTACATTCTTATTCTACCTGGCTTCGGTATTATCTCACACGTTGTAGCATACTACTCCAG
CAAAAAAGAATCATTCGGCTACATAGGTATAGTCTGAGCTATATTATCAATTGGTCTTCTTG
GTTTCATTGTTTGAGCCCACCATATATTCACTACAGACCTAAACGTTGACACACGAGCATAT
TTTACTTCAGCCACAATAATCATTGCTATTCCCACTGGCGTTAAAGTTTTCAGTTGATTGGC
TACCATGCACGGTGGCATCATTAAATGAGAAGCTCCAATACTATGAGCATTAGGCTTCATCT
TCTTATTTACCATCGGAGGCCTAACCGGCATTGTTCTTGCCAACTCCTCTATCGATATTGTT
CTTCACGATACCTACTACGTAGTTGCTCACTTCCACTACGTATTATCAATAGGAGCCGTCTT
TGCTATCATAGCCGGATTTGTCCACTGATTCCC

>Sa03

GTCTACATTCTTATTCTACCTGGCTTCGGTATTATCTCACACGTTGTAGCATACTACTCCAG
CAAAAAAGAATCATTCGGCTATATAGGTATAGTTTGAGCTATATTATCAATCGGTCTTCTTG
GTTTCATTGTTTGAGCCCACCATATATTCACCACAGACCTAAACGTTGACACACGAGCATAT
TTTACTTCAGCCACAATAATCATTGCTATTCCCACTGGCGTTAAAGTTTTCAGTTGACTGGC
TACCATGCACGGTGGTATTATTAAATGAGAAGCTCCAATACTATGAGCATTAGGCTTCATCT
TCTTATTTACCATCGGAGGCCTAACCGGCATTGTTCTTGCCAACTCCTCTATCGATATTGTT
CTTCACGATACCTACTACGTAGTAGCTCACTTCCACTACGTACTATCAATAGGAGCCGTCTT
TGCTATCATAGCTGGATTCGTCCACTGATTCCC

>Sa01

GTCTACATTCTTATTCTACCTGGCTTCGGTATTATCTCACACGTTGTAGCATACTACTCCAG
CAAAAAAGAATCATTCGGCTATATAGGTATAGTTTGAGCTATATTATCAATCGGTCTTCTTG
GTTTCATTGTTTGAGCCCACCATATATTCACCACAGACCTAAACGTTGACACACGAGCATAT
TTTACTTCAGCCACAATAATCATTGCTATTCCCACTGGCGTTAAAGTTTTCAGTTGACTGGC
TACCATGCACGGTGGCATCATTAAATGAGAAGCTCCAATACTATGAGCATTAGGCTTCATCT
TCTTATTTACCATCGGAGGCCTAACCGGCATTGTTCTTGCCAACTCCTCTATCGATATTGTT
CTTCACGATACCTACTACGTAGTAGCTCACTTCCACTACGTACTATCAATAGGAGCCGTCTT
TGCTATCATAGCTGGATTCGTCCACTGATTCCC

>Sa02

GTCTACATTCTTATTCTACCTGGCTTCTGTATTATTACACACGTTGGAGCATACTACTCCAG
CAAAAAAAAATCATTCGGCTATATAGGTATAGTTTGAGCTATACTATCAATCGGTCTTCTTG
GTTTCATTGTTTGAGCCCACCATATATTCACCACAGACCTAAACGTTGACACACGAGCATAT
TTTACTTCAGCCACAATAATCATTGCTATTCCCACTGGCGTTAAAGTTTTCAGTTGACTGGC
TACCATGCACGGTGGTATCATTAAATGAGAAGCTCCAATACTATGAGCATTAGGCTTCATCT
TCTTATTTACCATCGGAGGCCTAACCGGCATTGTTCTTGCCAACTCCTCTATCGATATTGTT
CTTCACGATACCTACTACGTAGTAGCTCACTTCCACTACGTACTATCAATAGGAGCCGTCTT
TGCTATCATAGCTGGATTCGTCCACTGATTCCC

>Ka05

GTCTACATTCTTATTCTACCTGGCTTCGGTATTATCTCACACGTTGTAGCATACTACTCCAG
CAAAAAAGAATCATTCGGCTACATAGGTATAGTCTGAGCTATATTATCAATTGGTCTTCTTG
GTTTCATTGTTTGAGCCCACCATATATTCACCACAGACCTAAACGTTGACACACGAGCATAT
TTTACTTCAGCCACAATAATCATTGCTATTCCCACTGGCGTTAAAGTTTTCAGTTGATTGGC
TACCATGCACGGTGGCATCATTAAATGAGAAGCTCCAATACTATGAGCATTAGGCTTCATCT
TCTTATTTACCATCGGAGGCCTAACCGGCATTGTTCTTGCCAACTCCTCTATCGATATTGTT
CTTCACGATACATACTACGTAGTTGCTCACTTCCACTACGTATTATCAATAGGAGCCGTCTT
TGCTATCATAGCCGGATTTGTCCACTGATTCCC

>Ka01

GTCTACATTCTTATTCTACCTGGCTTCGGTATTATCTCACACGTTGTAGCATACTACTCCAG
CAAAAAAGAATCGTTCGGCTACATAGGTATAGTCTGAGCTATATTATCAATTGGCCTTCTTG
GTTTCATTGTTTGAGCCCACCATATATTCACCACAGACCTAAACGTTGACACACGAGCATAT
TTTACTTCAGCCACAATAATCATTGCTATTCCCACTGGCGTTAAAGTTTTCAGTTGATTGGC
TACCATGCACGGTGGCATCATTAAATGAGAAGCTCCAATACTATGAGCATTAGGCTTCATCT
TCTTATTTACCATCGGAGGCCTAACCGGCATTGTTCTTGCCAACTCCTCTATCGATATTGTT
CTTCACGATACCTACTACGTAGTTGCTCACTTCCACTACGTATTATCAATAGGAGCCGTCTT
TGCTATCATAGCCGGATTTGTCCACTGATTCCC

>Ka06

GTCTACATTCTTATTCTACCTGGCTTCGGTATTATCTCACACGTTGTAGCATACTACTCCAG
CAAAAAAGAATCATTCGGCTACATAGGTATAGTCTGAGCTATATTATCAATTGGTCTTCTTG
GTTTCATTGTTTGAGCCCACCATATATTCACCACAGACCTAAACGTTGACACACGAGCATAT
TTTACTTCAGCCACAATAATCATTGCTATTCCCACTGGCGTTAAAGTTTTCAGTTGATTGGC
TACCATGCACGGTGGCATCATTAAATGAGAAGCTCCAATACTATGAGCATTAGGCTTCATCT
TCTTATTTACCATCGGAGGCCTAACCGGCATTGTTCTTGCCAACTCCTCTATCGATATTGTT
CTTCACGATACATACTACGTAGTTGCTCACTTCCACTACGTATTATCAATAGGAGCCGTCTT
TGCTATCATAGCCGGATTTGTCCACTGATTCCC

>Ka03

GTCTACATTCTTATTCTACCTGGCTTCGGTATTATCTCACACGTTGTAGCATACTACTCCAG
CAAAAAAGAATCATTCGGCTACATAGGTATAGTCTGAGCTATATTATCAATTGGTCTTCTTG

GTTTCATTGTTTGAGCCCACCATATATTCACCACAGACCTAAACGTTGACACACGAGCATAT
TTTACTTCAGCCACAATAATTATTGCTATTCCCACTGGCGTTAAAGTTTTCAGTTGATTGGCT
ACCATGCACGGTGGCATCATTAAATGAGAAGCTCCAATACTATGAGCATTAGGCTTCATCTT
CTTATTTACCATCGGAGGCCTAACCGGCATTGTTCTTGCCAACTCCTCTATTGATATTGTTC
TTCACGATACCTACTACGTAGTTGCTCACTTCCACTACGTATTATCAATAGGAGCCGTCTTT
GCTATCATAGCCGGATTTGTCCACTGATTCCC

>Ka04

GTCTACATTCTTATTCTACCTGGCTTCGGTATTATCTCACACGTTGTAGCATACTACTCCAG
CAAAAAAGAATCATTCGGCTACATAGGTATAGTCTGAGCTATATTATCAATTGGTCTTCTTG
GTTTCATTGTTTGAGCCCACCATATATTCACCACAGACCTAAACGTTGACACACGAGCATAT
TTTACTTCAGCCACAATAATCATTGCTATTCCCACTGGCGTTAAAGTTTTCAGTTGATTGGC
TACCATGCATGGTGGCATCATTAAATGAGAAGCTCCAATACTATGAGCATTAGGCTTCATCT
TCTTATTTACCATCGGAGGCCTAACCGGCATTGTTCTTGCCAACTCCTCTATCGATATTGTT
CTTCACGATACCTACTACGTAGTTGCTCACTTCCACTACGTATTATCAATAGGAGCTGTCTT
TGCTATCATAGCCGGATTTGTCCACTGATTCCC

>Ka02

GTCTACATTCTTATTCTACCTGGCTTCGGTATTATCTCACACGTTGTAGCATACTACTCCAG
CAAAAAAGAATCATTCGGCTACATAGGTATAGTCTGAGCTATATTATCAATTGGCCTTCTTG
GTTTCATTGTTTGAGCCCACCATATATTCACCACAGACCTAAACGTTGACACACGAGCATAT
TTTACTTCAGCCACAATAATCATTGCTATTCCCACTGGCGTTAAAGTTTTCAGTTGATTGGC
TACCATGCACGGTGGCATCATTAAATGAGAAGCTCCAATACTATGAGCATTAGGCTTCATCT
TCTTATTTACCATCGGAGGCCTAACCGGCATTGTTCTTGCCAACTCCTCTATCGATATTGTT
CTTCACGATACCTACTACGTAGTTGCTCACTTCCACTACGTATTATCAATAGGAGCCGTCTT
TGCTATCATAGCCGGATTTGTCCACTGATTCCC

>Mu06

GTCTACATTCTTATTCTACCTGGCTTCGGTATTATCTCACACGTTGTAGCATACTACTCCAG
CAAAAAAGAATCATTCGGCTATATGGGCATAGTCTGAGCTATATTATCAATTGGCCTTCTTG
GTTTTATTGTTTGAGCTCACCATATATTCACCACAGACCTAAACGTTGACACACGAGCATAC
TTTACTTCAGCCACAATAATCATTGCTATTCCCACTGGCGTTAAAGTTTTCAGTTGACTGGC
TACCATGCACGGTGGTATCATTAAATGAGAAGCTCCAATACTATGAGCATTAGGCTTCATCT
TCTTATTTACCATCGGAGGCCTAACCGGCATTGTTCTTGCCAACTCCTCTATCGATATTGTT
CTTCATGACACCTACTACGTGGTTGCTCACTTCCACTACGTACTATCAATAGGAGCCGTCTT
TGCTATCATAGCTGGATTCGTCCACTGATTCCC

>Ku04

GTCTACCTTCTTATTCTACCTGGCTTCTGTATTATTTCACACGTTGTAGCATACTACTCCAGC
AAAAAAAAACCATTCTGCTATATGGGGATATTCTGAGCCATATTATCAATTGGTCTCCTTGG
TTTTATTGTGTGAGCCCACCACATATTCACTACAGATCTAAACGTTGACACACGAGCATATT
TTACTTCAGCCACAATAATCATTGCTATTCCCACTGGTGTTAAAGTTTTTAGTTGGCTGGCC
ACCATGCACGGCGGTGTTATTAAATGAGAAGCTCCAATATTGTGAGCATTAGGCTTCATTTT

CTTATTCACCATCGGAGGGCTAACCGGCATTGTTCTCGCCAACTCCTCAATCGATATTGTT
CTTCACGACACCTACTACGTAGTCGCCCATTTCCACTATGTATTATCAATAGGAACCGTCTT
TGCTATCATAGCCGGATTTGTTCACTGATTCCC

>Mu01

GTCTACCTTCTTATTCTACCTGGCTTCTGTATTATCTCACACGTTGTAGCATACTACTCCAG
CAAAAAAAAATCATTCTGCTATATGGGCATATTCTGAGATATATTATCAATTGTCCTTCTTGG
TTTTATTGTTTGAGCTCACCATATATTCACCACAGACCTAAACGTTGACACACGAGCATACT
TTACTTCAGCCACAATAATCATTGCTATTCCCACTGGCGTTAAAGTTTTTAGTTGACTGGCT
ACCATGCACGGTGGTATCATTAAATGAGAAGCTCCAATACTATGAGCATTAGGCTTCATCTT
CTTATTTACCATCGGAGGCCTAACCGGCATTGTTCTTGCCAACTCCTCTATCGATATTGGTC
TTCATGACACCTACTACGTGGTTGCTCACTTCCACTACGTACTATCAATAGGAACCGTCTTT
GCTATCATAACTGGATTCCTCCACTGATTCCC

>Mu02

GTCTACATTCTTATTCTACCTGGCTTCTGTATTATCTCACACGTTGTAGCATACTACTCCAG
CAAAAAAGAATCATTCTGCTATATGGGCATATTCTGAGCTATATTATCAATTGGCCTTCTTG
GTTTTATTGTTTGAGCTCACCATATATTCACCACAGACCTAAACGTTGACACACGAGCATAC
TTTACTTCAGCCACAATAATCATTGCTATTCCCACTGGCGTTAAAGTTTTTAGTTGACTGGC
TACCATGCACGGTGGTATCATTAAATGAGAAGCTCCAATACTATGAGCATTAGGCTTCATCT
TCTTATTTACCATCGGAGGCCTAACCGGCATTGTTCTTGCCAACTCCTCTATCGATATTGTT
CTTCATGACACCTACTACGTGGTTGCTCACTTCCACTACGTACTATCAATAGGAGCCGTCTT
TGCTATCATAGCTGGATTCCTCCACTGATTCCC

>Bt09

CCCAGTCTACATTCTTATTCTACCTGGCTTCGGTATTATCTCACACGTTGTAGCATACTACT
CCAGCAAAAAATCATTCGGCTACATTATAGTCTGAGCTATATTATCAATTGGTCTTCTTGGTT
TCATTGTTTGAGCCCACCATATATTCACCACCCTAAACGTTGACACACGAGCATATTTTACT
TCAGCCACAATAATCATTGCTATTCCCACTGGCGTAGTTTTTTGATTGGCTACCATGCATGG
TGGCATCATATGAGCTCCAATACTATGAGCATTCTTCATCTTCTTATTTACCATCGGCCTAA
CCGGCATTGTTCTTGCCAACTCCTCTATCGATATTGTTCTTCACGATACCTACTACGTAGTT
GCTCACTTCCACTACGTATTATCAATAGCTGTCTTTGCTATCATAGCCGGATTTGTCCACTG
ATTCCCGTTATTTACTGGA

>Bt08

CCCAGTCTACATTCTTATTCTACCTGGCTTCGGTATTATCTCACACGTTGTAGCATACTACT
CCAGCAAAAAATCATTCGGCTACATTATAGTCTGAGCTATATTATCAATTGGTCTTCTTGGTT
TCATTGTTTGAGCCCACCATATATTCACCACCCTAAACGTTGACACACGAGCATATTTTACT
TCAGCCACAATAATCATTGCTATTCCCACTGGCGTAGTTTTTTGATTGGCTACCATGCACGG
TGGCATCATATGAGCTCCAATACTATGAGCATTTTTCATCTTCTTATTTACCATCGGCCTAAC
CGGCATTGTTCTTGCCAACTCCTCTATCGATATTGTTCTTCACGATACCTACTACGTAGTTG
CTCACTTCCACTACGTATTATCAATAGCTGTCTTTGCTATCATAGCCGGATTTGTCCACTGA
TTCCCGTTATTTACTGGA

>Pa07

TCCAGTCTACATTCTTATTCTACCTGGCTTCGGTATTATTTCACACGTTGTAGCATACTACTC
CAGCAAAAAACCATTCGGCTATATCATAGTCTGAGCCATATTATCAATTGGTCTCCTTGGTT
TTATTGTCTGAGCCCACCACATATTCACTACTCTAAACGTTGACACACGAGCATATTTTACT
TCAGCCACAATAATCATTGCTATTCCCACTGGTGTAGTTTTTTGGCTGGCCACCATGCACG
GCGGTGTTATATGAGCTCCAATATTGTGAGCATTCTTCATTTTCTTATTCACCATCGGCCTA
ACCGGCATTGTTCTCGCCAACTCCTCAATCGATATTGTTCTTCACGACACCTACTACGTAGT
CGCCCATTTCCACTATGTATTATCAATAGCCGTCTTTGCTATCATAGCCGGATTTGTTCACT
GATTCCCGTTATTTACTGGA

>Pa08

TCCAGTCTACATTCTTATTCTACCTGGCTTCGGTATTATTTCACACGTTGTAGCATACTACTC
CAGCAAAAAACCATTCGGCTATATCATAGTCTGAGCCATATTATCAATTGGTCTCCTTGGTT
TTATTGTCTGAGCCCACCACATATTCACTACTCTAAACGTTGACACACGAGCATATTTTACT
TCAGCCACAATAATCATTGCTATTCCCACTGGTGTAGTTTTTTGGCTGGCCACCATGCACG
GCGGTGTTATATGAGCTCCAATATTGTGAGCATTCTTCATTTTCTTATTCACCATCGGCCTA
ACCGGCATTGTTCTCGCCAACTCCTCAATCGATATTGTTCTTCACGACACCTACTACGTAGT
CGCCCATTTCCACTATGTATTATCAATAGCCGTCTTTGCTATCATAGCCGGATTTGTTCACT
GATTCCCGTTATTTACTGGA

>Pa09

TCCAGTCTACATTCTTATTCTACCTGGCTTCGGTATTATTTCACACGTTGTAGCATACTACTC
CAGCAAAAAACCATTCGGCTATATCATAGTCTGAGCCATATTATCAATTGGTCTCCTTGGTT
TTATTGTCTGAGCCCACCACATATTCACTACTCTAAACGTTGACACACGAGCATATTTTACT
TCAGCCACAATAATCATTGCTATTCCCACTGGTGTAGTTTTTTGGCTGGCCACCATGCACG
GCGGTGTTATATGAGCTCCAATATTGTGAGCATTCTTCATTTTCTTATTCACCATCGGCCTA
ACCGGCATTGTTCTCGCCAACTCCTCAATCGATATTGTTCTTCACGACACCTACTACGTAGT
CGCCCATTTCCACTATGTATTATCAATAGCCGTCTTTGCTATCATAGCCGGATTTGTTCACT
GATTCCCGTTATTTACTGGA

>Eng02

TCCAGTCTACATTCTTATTCTACCTGGCTTCGGTATTATTTCACACGTTGTAGCATACTACTC
CAGCAAAAAACCATTCGGCTATATCATAGTCTGAGCCATATTATCAATTGGTCTCCTTGGTT
TTATTGTCTGAGCCCACCACATATTCACTACTCTAAACGTTGACACACGAGCATATTTTACT
TCAGCCACAATAATCATTGCTATTCCCACTGGTGTAGTTTTTTGACTGGCCACCATGCACG
GCGGTGTTATATGAGCTCCAATATTGTGAGCATTCTTCATTTTCTTATTCACCATCGGCCTA
ACCGGCATTGTTCTCGCCAACTCCTCAATCGATATTGTTCTTCACGACACCTACTACGTAGT
CGCCCATTTCCACTATGTATTATCAATAGCCGTCTTTGCTATCATAGCCGGATTTGTTCACT
GATTCCCGTTATTTACTGGA

>Eng01

TCCAGTCTACATTCTTATTCTACCTGGCTTCGGTATTATTTCACACGTTGTAGCATACTACTC
CAGCAAAAAACCATTCGGCTATATCATAGTCTGAGCCATATTATCAATTGGTCTCCTTGGTT
TTATTGTCTGAGCCCACCACATATTCACTACTCTAAACGTTGACACACGAGCATATTTTACT
TCAGCCACAATAATCATTGCTATTCCCACTGGTGTAGTTTTTTGACTGGCCACCATGCACG
GCGGTGTTATATGAGCTCCAATATTGTGAGCATTCTTCATTTTCTTATTCACCATCGGCCTA
ACCGGCATTGTTCTCGCCAACTCCTCAATCGATATTGTTCTTCACGACACCTACTACGTAGT
CGCCCATTTCCACTATGTACTATCAATAGCCGTCTTTGCTATCATAGCCGGATTTGTTCACT
GATTCCCGTTATTTACTGGA

>Eng03

TCCAGTCTACATTCTTATTCTACCTGGCTTCGGTATTATTTCACACGTTGTAGCATACTACTC
CAGCAAAAAACCATTCGGCTATATCATAGTCTGAGCCATATTATCAATTGGTCTCCTTGGTT
TTATTGTCTGAGCCCACCACATATTCACTACTCTAAACGTTGACACACGAGCATATTTTACT
TCAGCCACAATAATCATTGCTATTCCCACTGGTGTAGTTTTTTGACTGGCCACCATGCACG
GCGGTGTTATATGAGCTCCAATATTGTGAGCATTCTTCATTTTCTTATTCACCATCGGCCTA
ACCGGCATTGTTCTCGCCAACTCCTCAATCGATATTGTTCTTCACGACACCTACTACGTAGT
CGCCCATTTCCACTATGTACTATCAATAGCCGTCTTTGCTATCATAGCCGGATTTGTTCACT
GATTCCCGTTATTTACTGGT

>Ga01

TCCAGTCTACATTCTTATTCTACCTGGCTTCGGTATTATTTCACACGTTGTAGCATACTACTC
CAGCAAAAAACCATTCGGCTATATCATAGTCTGAGCCATATTATCAATTGGTCTCCTTGGTT
TTATTGTCTGAGCCCACCACATATTCACTACTCTAAACGTTGACACACGAGCATATTTTACT
TCAGCCACAATAATCATTGCTATTCCCACTGGTGTAGTTTTTTGGCTGGCCACCATGCACG
GCGGTGTTATATGAGCTCCAATATTGTGAGCACTCTTCATTTTCTTATTCACCATCGGCCTA
ACCGGCATTGTTCTCGCCAACTCCTCAATCGATATTGTTCTTCACGACACCTACTACGTAGT
CGCCCATTTCCACTATGTATTATCAATAGCCGTCTTTGCTATCATAGCCGGATTTGTTCACT
GATTCCCGTTATTTACTGGA

>Ga02

TCCAGTCTACATTCTTATTCTACCTGGCTTCGGTATTATTTCACACGTTGTAGCATACTACTC
CAGCAAAAAACCATTCGGCTATATCATAGTCTGAGCCATATTATCAATTGGTCTCCTTGGTT
TTATTGTCTGAGCCCACCACATATTCACTACTCTAAACGTTGACACACGAGCATATTTTACT
TCAGCCACAATAATCATTGCTATTCCCACTGGTGTAGTTTTTTGGCTGGCCACCATGCACG
GCGGTGTTATATGAGCTCCAATATTGTGAGCATTCTTCATTTTCTTATTCACCATCGGCCTA
ACCGGCATTGTTCTCGCCAACTCCTCAATCGATATTGTTCTTCACGACACCTACTACGTAGT
CGCCCATTTCCACTATGTATTATCAATAGCCGTCTTTGCTATCATAGCCGGATTTGTTCACT
GATTCCCGTTATTTACTGGA

>Ga03

TCCAGTCTACATTCTTATTCTACCTGGCTTCGGTATTATTTCACACGTTGTAGCATACTACTC
CAGCAAAAAACCATTCGGCTATATCATAGTCTGAGCCATATTATCAATTGGTCTCCTTGGTT

TTATTGTCTGAGCCCACCACATATTCACTACTCTAAACGTTGACACACGAGCATATTTTACT
TCAGCCACAATAATCATTGCTATTCCCACTGGTGTAGTTTTTTGGCTGGCCACCATGCACG
GCGGTGTTATATGAGCTCCAATATTGTGAGCACTCTTCATTTTCTTATTCACCATCGGCCTA
ACCGGCATTGTTCTCGCCAACTCCTCAATCGATATTGTTCTTCACGACACCTACTACGTAGT
CGCCCATTTCCACTATGTATTATCAATAGCCGTCTTTGCTATCATAGCCGGATTTGTTCACT
GATTCCCGTTATTTACTGGA

>Ka07

CCCAGTCTACATTCTTATTCTACCTGGCTTCGGTATTATCTCACACGTTGTAGCATACTACT
CCAGCAAAAAATCATTCGGCTACATTATAGTCTGAGCTATATTATCAATTGGTCTTCTTGGTT
TTATTGTTTGAGCCCACCATATATTCACCACCCTAAACGTTGACACACGAGCATATTTTACT
TCAGCCACAATAATCATTGCTATTCCCACTGGCGTAGTTTTTTGATTGGCTACCATGCACGG
TTGCATCATATGAGCTCCAATACTATGAGCATTCTTCATCTTCTTATTTACCATCGGCCTAAC
CGGCATTGTTCTTGCCAACTCCTCTATCGATATTGTTCTTCACGATACCTACTACGTAGTTG
CTCACTTCCACTACGTATTATCAATAGCCGTCTTTGCTATCATAGCCGGATTTGTCCACTGA
TTCCCGTTATTTACTGGa

>Ka08

CCCAGTCTACATTCTTATTCTACCTGGCTTCGGTATTATCTCACACGTTGTAGCATACTACT
CCAGCAAAAAATCATTCGGCTATATCATAGTCTGAGCTATATTATCAATTGGCCTTCTTGGT
TTTATTGTTTGAGCTCACCATATATTCACCACCCTAAACGTTGACACACGAGCATACTTTAC
TTCAGCCACAATAATCATTGCTATTCCCACTGGCGTAGTTTTTTGACTGGCTACCATGCACG
GTGGTATCATATGAGCTCCAATACTATGAGCATTCTTCATCTTCTTATTTACCATCGGCCTA
ACCGGCATTGTTCTTGCCAACTCCTCTATCGATATTGTTCTTCATGACACCTACTACGTGGT
TGCTCACTTCCACTACGTACTATCAATAGCCGTCTTTGCTATCATAGCTGGATTCGTCCACT
GATTCCCGTTATTTACTGGA

>Ka09

CCCAGTCTACATTCTTATTCTACCTGGCTTCGGTATTATCTCACACGTTGTAGCATACTACT
CCAGCAAAAAATCATTCGGCTACATTATAGTCTGAGCTATATTATCAATTGGCCTTCTTGGT
TTCATTGTTTGAGCCCACCATATATTCACCACCCTAAACGTTGACACACGAGCATATTTTAC
TTCAGCCACAATAATCATTGCTATTCCCACTGGCGTAGTTTTTTGATTGGCTACCATGCACG
GTGGCATCATATGAGCTCCAATACTATGAGCATTCTTCATCTTCTTATTTACCATCGGCCTA
ACCGGCATTGTTCTTGCCAACTCCTCTATCGATATTGTTCTTCACGATACCTACTACGTAGT
TGCTCACTTCCACTACGTATTATCAATAGCCGTCTTTGCTATCATAGCCGGATTTGTCCACT
GATTCCCGTTATTTACTGGA

>Pa10

TCCAGTCTACATTCTTATTCTACCTGGCTTCGGTATTATTTCACACGTTGTAGCATACTACTC
CAGCAAAAAACCATTCGGCTATATCATAGTCTGAGCCATATTATCAATTGGTCTCCTTGGTT
TTATTGTCTGAGCCCACCACATATTCACTACTCTAAACGTTGACACACGAGCATATTTTACT
TCAGCCACAATAATCATTGCTATTCCCACTGGTGTAGTTTTTTGGCTGGCCACCATGCACG
GCGGTGTTATATGAGCTCCAATATTGTGAGCATTCTTCATTTTCTTATTCACCATCGGCCTA

ACCGGCATTGTTCTCGCCAACTCCTCAATCGATATTGTTCTTCACGACACCTACTACGTAGT
CGCCCATTTCCACTATGTATTATCAATAGCCGTCTTTGCTATCATAGCCGGATTTGTTCACT
GATTCCCGTTATTTACTGGA

>Pa11

TCCAGTCTACATTCTTATTCTACCTGGCTTCGGTATTATTTCACACGTTGTAGCATACTACTC
CAGCAAAAAACCATTCGGCTATATCATAGTCTGAGCCATATTATCAATTGGTCTCCTTGGTT
TTATTGTCTGAGCCCACCACATATTCACTACTCTAAACGTTGACACACGAGCATATTTTACT
TCAGCCACAATAATCATTGCTATTCCCACTGGTGTAGTTTTTTGGCTGGCCACCATGCACG
GCGGTGTTATATGAGCTCCAATATTGTGAGCATTCTTCATTTTCTTATTCACCATCGGCCTA
ACCGGCATTGTTCTCGCCAACTCCTCAATCGATATTGTTCTTCACGACACCTACTACGTAGT
CGCCCATTTCCACTATGTATTATCAATAGCCGTCTTTGCTATCATAGCCGGATTTGTTCACT
GATTCCCGTTATTTACTGGA

>Ku06

TCCAGTCTACATTCTTATTCTACCTGGCTTCGGTATTATTTCACACGTTGTAGCATACTACTC
CAGCAAAAAACCATTCGGCTATATCATAGTCTGAGCCATATTATCAATTGGTCTCCTTGGTT
TTATTGTCTGAGCCCACCACATATTCACTACTCTAAACGTTGACACACGAGCATATTTTACT
TCAGCCACAATAATCATTGCTATTCCCACTGGTGTAGTTTTTTGGCTGGCCACCATGCACG
GCGGTGTTATATGAGCTCCAATATTGTGAGCATTCTTCATTTTCTTATTCACCATCGGCCTA
ACCGGCATTGTTCTCGCCAACTCCTCAATCGATATTGTTCTTCACGACACCTACTACGTAGT
CGCCCATTTCCACTATGTATTATCAATAGCCGTCTTTGCTATCATAGCCGGATTTGTTCACT
GATTCCCGTTATTTACTGGA

>Ku07

TCCAGTCTACATTCTTATTCTACCTGGCTTCGGTATTATTTCACACGTTGTAGCATACTACTC
CAGCAAAAAACCATTCGGCTATATCATAGTCTGAGCCATATTATCAATTGGTCTCCTTGGTT
TTATTGTCTGAGCCCACCACATATTCACTACTCTAAACGTTGACACACGAGCATATTTTACT
TCAGCCACAATAATCATTGCTATTCCCACTGGTGTAGTTTTTTGGCTGGCCACCATGCACG
GCGGTGTTATATGAGCTCCAATATTGTGAGCATTCTTCATTTTCTTATTCACCATCGGCCTA
ACCGGCATTGTTCTCGCCAACTCCTCAATCGATATTGTTCTTCACGACACCTACTACGTAGT
CGCCCATTTCCACTATGTATTATCAATAGCCGTCTTTGCTATCATAGCCGGATTTGTTCACT
GATTCCCGTTATTTACTGGA

>Mulu01

CCCAGTCTACATTCTTATTCTACCTGGCTTCGGTATTATCTCACACGTTGTAGCATACTACT
CCAGCAAAAAATCATTCGGCTATATCATAGTCTGAGCTATATTATCAATTGGTCTTCTTGGTT
TTATTGTTTGAGCTCACCATATATTCACCACCCTAAACGTTGACACACGAGCATATTTTACTT
CAGCCACAATAATCATTGCTATTCCCACTGGCGTAGTTTTTTGACTGGCTACCATGCACGG
TGGTATCATATGAGCTCCAATACTATGAGCATTCTTCATCTTCTTATTTACCATCGGCCTAAC
CGGCATTGTTCTTGCCAACTCCTCTATCGATATTGTTCTTCATGACACCTACTACGTAGTTG
CTCACTTCCACTACGTACTATCAATAGCCGTCTTTGCTATCATAGCTGGATTCGTCCACTGA
TTCCCGTTATTTACTGGA

>Mulu02

CCCAGTCTATATTCTTATTCTACCTGGCTTCGGTATTATCTCACACGTTGTAGCATACTACT
CCAGCAAAAAATCATTCGGCTATATCATAGTCTGAGCTATATTATCAATTGGCCTTCTTGGT
TTTATTGTTTGAGCTCACCATATATTCACCACCCTAAACGTTGACACACGAGCATACTTTAC
TTCAGCCACAATAATCATTGCTATTCCCACTGGCGTAGTTTTTTGACTGGCTACCATGCACG
GTGGTATCATATGAGCTCCAATACTATGAGCATTCTTCATCTTCTTATTTACCATCGGCCTA
ACCGGCATTGTTCTTGCCAACTCCTCTATCGATATTGTTCTTCATGACACCTACTACGTGGT
TGCTCACTTCCACTACGTACTATCAATAGCCGTCTTTGCTATCATAGCTGGATTCGTCCACT
GATTCCCGTTATTTACTGGA

>Mulu05

CCCAGTCTACATTCTTATTCTACCTGGCTTCGGTATTATCTCACACGTTGTAGCATACTACT
CCAGCAAAAAATCATTCGGCTATATCATAGTCTGAGCTATATTATCAATTGGCCTTCTTGGT
TTTATTGTTTGAGCTCACCATATATTCACCACCCTAAACGTTGACACACGAGCATACTTTAC
TTCAGCCACAATAATCATTGCTATTCCCACTGGCGTAGTTTTTTGACTGGCTACCATGCACG
GTGGTATCATATGAGCTCCAATACTATGAGCATTCTTCATCTTCTTATTTACCATCGGCCTA
ACCGGCATTGTTCTTGCCAACTCCTCTATCGATATTGTTCTTCATGACACCTACTACGTGGT
TGCTCACTTCCACTACGTACTATCAATAGCCGTCTTTGCTATCATAGCTGGATTCGTCCACT
GATTCCCGTTATTTACTGGA

>Mulu07

CCCAGTCTACATTCTTATTCTACCTGGCTTCGGTATTATCTCACACGTTGTAGCATACTACT
CCAGCAAAAAATCATTCGGCTATATCATAGTCTGAGCTATATTATCAATTGGCCTTCTTGGT
TTTATTGTTTGAGCTCACCATATATTCACCACCCTAAACGTTGACACACGAGCATACTTTAC
TTCAGCCACAATAATCATTGCTATTCCCACTGGCGTAGTTTTTTGACTGGCTACCATGCACG
GTGGTATCATATGAGCTCCAATACTATGAGCATTCTTCATCTTCTTATTTACCATCGGCCTA
ACCGGCATTGTTCTTGCCAACTCCTCTATCGATATTGTTCTTCATGACACCTACTACGTGGT
TGCTCACTTCCACTACGTACTATCAATAGCCGTCTTTGCTATCATAGCTGGATTCGTCCACT
GATTCCCGTTATTTACTgGA

>Seb01

TCCAGTCTACATTCTTATTCTACCTGGCTTCGGTATTATTTCACACGTTGTAGCATACTACTC
CAGCAAAAAACCATTCGGCTATATCATAGTCTGAGCCATATTATCAATTGGTCTCCTTGGTT
TTATTGTCTGAGCCCACCACATATTCACTACTCTAAACGTTGACACACGAGCATATTTTACT
TCAGCCACAATAATCATTGCTATTCCCACTGGTGTAGTTTTTTGGCTGGCCACCATGCACG
GCGGTGTTATATGAGCTCCAATATTGTGAGCATTCTTCATTTTCTTATTCACCATCGGCCTA
ACCGGCATTGTTCTCGCCAACTCCTCAATCGATATTGTTCTTCACGACACCTACTACGTAGT
CGCCCATTTCCACTATGTACTATCAATAGCCGTCTTTGCTATCATAGCCGGATTTGTTCACT
GATTCCCGTTATTTACTGGA

>Seb02

TCCAGTCTACATTCTTATTCTACCTGGCTTCGGTATTATTTCACACGTTGTAGCATACTACTC
CAGCAAAAAACCATTCGGCTATATCATAGTCTGAGCCATATTATCAATTGGTCTCCTTGGTT
TTATTGTCTGAGCCCACCACATATTCACTACTCTAAACGTTGACACACGAGCATATTTTACT
TCAGCCACAATAATCATTGCTATTCCCACTGGTGTAGTTTTTTGGCTGGCCACCATGCACG
GCGGTGTTATATGAGCTCCAATATTGTGAGCATTCTTCATTTTCTTATTCACCATCGGCCTA
ACCGGCATTGTTCTCGCCAACTCCTCAATCGATATTGTTCTTCACGACACCTACTACGTAGT
CGCCCATTTCCACTATGTACTATCAATAGCCGTCTTTGCTATCATAGCCGGATTTGTTCACT
GATTCCCGTTATTTACTGGA

>Wil03

CCCAGTCTACATTCTTATTCTACCTGGCTTCGGTATTATCTCACACGTTGTAGCATACTACT
CCAGCAAAAAATCATTCGGCTATATCATAGTCTGAGCTATATTATCAATTGGCCTTCTTGGT
TTTATTGTTTGAGCTCACCATATATTCACCACCCTAAACGTTGACACACGAGCATACTTTAC
TTCAGCCACAATAATCATTGCTATTCCCACTGGCGTAGTTTTTTGACTGGCTACCATGCACG
GTGGTATCATATGAGCTCCAATACTATGAGCATTCTTCATCTTCTTATTTACCATCGGCCTA
ACCGGCATTGTTCTTGCCAACTCCTCTATCGATATTGTTCTTCATGACACCTACTACGTGGT
TGCTCACTTCCACTACGTACTATCAATAGCCGTCTTTGCTATCATAGCTGGATTCGTCCACT
GATTCCCGTTATTTACTGGA

>Wil01

CCCAGTCTACATTCTTATTCTACCTGGCTTCGGTATTATCTCACACGTTGTAGCATACTACT
CCAGCAAAAAATCATTCGGCTATATCATAGTCTGAGCTATATTATCAATTGGCCTTCTTGGT
TTTATTGTTTGAGCTCACCATATATTCACCACCCTAAACGTTGACACACGAGCATACTTTAC
TTCAGCCACAATAATCATTGCTATCCCCACTGGCGTAGTTTTTTGACTGGCTACCATGCACG
GTGGTATCATATGAGCTCCAATACTATGAGCATTCTTCATCTTCTTATTTACCATCGGCCTA
ACCGGCATTGTTCTTGCCAACTCCTCTATCGATATTGTTCTTCATGACACCTACTACGTGGT
TGCTCACTTCCACTACGTACTATCAATAGCCGTCTTTGCTATCATAGCTGGATTCGTCCACT
GATTCCCGTTATTTACTGGA

>Wil02

CCCAGTCTACATTCTTATTCTACCTGGCTTCGGTATTATCTCACACGTTGTAGCATACTACT
CCAGCAAAAAATCATTCGGCTATATCATAGTCTGAGCTATATTATCAATTGGCCTTCTTGGT
TTTATTGTTTGAGCTCACCATATATTCACCACCCTAAACGTTGACACACGAGCATACTTTAC
TTCAGCCACAATAATCATTGCTATTCCCACTGGCGTAGTTTTTTGACTGGCTACCATGCACG
GTGGTATCATATGAGCTCCAATACTATGAGCATTCTTCATCTTCTTATTTACCATCGGCCTA
ACCGGCATTGTTCTTGCCAACTCCTCTATCGATATTGTTCTTCATGACACCTACTACGTGGT
TGCTCACTTCCACTACGTACTATCAATAGCCGTCTTTGCTATCATAGCTGGATTCGTCCACT
GATTCCCGTTATTTACTGGA

>Wil04

CCCAGTCTACATTCTTATTCTACCTGGCTTCGGTATTATCTCACACGTTGTAGCATACTACT
CCAGCAAAAAATCATTCGGCTATATCATAGTCTGAGCTATATTATCAATTGGCCTTCTTGGT

TTTATTGTTTGAGCTCACCATATATTCACCACCCTAAACGTTGACACACGAGCATACTTTAC
TTCAGCCACAATAATCATTGCTATTCCCACTGGCGTAGTTTTTTGACTGGCTACCATGCACG
GTGGTATCATATGAGCTCCAATACTATGAGCATTCTTCATCTTCTTATTTACCATCGGCCTA
ACCGGCATTGTTCTTGCCAACTCCTCTATCGATATTGTTCTTCATGACACCTACTACGTGGT
TGCTCACTTCCACTACGTACTATCAATAGCCGTCTTTGCTATCATAGCTGGATTCGTCCACT
GATTCCCGTTATTTACTGGA

List of aligned haplotypes sequences

Hap_1 ----GTCTACATTCTTATTCTACCTGGCTTCGGTATTATTTCACACGTTGTAGC-
TACTACTCCAGCAAAAAA---CCAT [80]

Hap_2 ----..-
..................---.... [80]

Hap_3 ----..-
..................---.... [80]

Hap_4 ----.............................A....................-
..................---.... [80]

Hap_5 ----...T..-
..................---.... [80]

Hap_6 ----..-
..................---.... [80]

Hap_7 ----..-
..................---.... [80]

Hap_8 ----..-
..................---.... [80]

Hap_9 ----..-
..................---.... [80]

Hap_10 ----......C....................T......................-
..................---.... [80]

Hap_11 ----..-
..................---.... [80]

Hap_12 ----..-
..................---.... [80]

Hap_13 ----..-
..................---.... [80]

Hap_14 ----..-
..................---.... [80]

Hap_15 ----..-
..................---.... [80]

Hap_16 ----...................................C..............-
..................---T... [80]

Hap_17 ----...................................C..............-
..................---T.G. [80]

Hap_18 ----...................................C..............-
..................---T... [80]

Hap_19 ----...................................C..............-
..................---T... [80]

Hap_20 ----...................................C..............-
..................---T... [80]

Hap_21 ----...................................C..............-
..................---T... [80]

Hap_22 ----...................................C..............-
..................---T... [80]

Hap_23 ----...................................C..............-
..................---T... [80]

Hap_24 ----...................................C..............-
..................---T... [80]

Hap_25 ----...................................C..............-
..................---T... [80]

Hap_26 ----...........................T........A.........G...-
..................---T... [80]

Hap_27 ----...................................C..............-
..................---T... [80]

Hap_28 ----......C....................T.......C..............-
..................---T... [80]

Hap_29 ----...........................T.......C..............-
..................---T... [80]

Hap_30 ----...................................C..............-
..................---T... [80]

Hap_31 ----...................................C..............-
..................---T... [80]

Hap_32 ----...................................C..............-
..................---T... [80]

Hap_33 ----...................................C..............-
..................---T... [80]

Hap_34 ----...................................C..............-
..................---T... [80]

Hap_35 ----.....T.............................C..............-
..................---T... [80]

Hap_36 ----...................................C..............-
..................---T... [80]

Hap_37 ----...................................C..............-
..................---T... [80]

Hap_1 TCGGCTATAT---CATAGTCTGAGCCATATTATCAATTGGTC-
CCTTGGTTTTATTGTCTGAGCCCACCACATATTCACT [160]

```
Hap_2  ..........---.............................-
..................................... [160]

Hap_3  ..........---.............................-
..................................... [160]

Hap_4  ..........---.............................-
T.................................... [160]

Hap_5  ..........---.............................-
..................................... [160]

Hap_6  ..........---.............................-
..................................... [160]

Hap_7  ..........---.............................-
..................................... [160]

Hap_8  ..........---......T......................-
..................................... [160]

Hap_9  ..........---.............................-
.G.C.C....................CA......... [160]

Hap_10 ..T.......---G...T........................-
...............G..................... [160]

Hap_11 ..........---.............................-
..................................... [160]

Hap_12 ..........---.............................-
..................................... [160]

Hap_13 ..........---.............................-
..................................... [160]

Hap_14 ..........---.............................-
..................................... [160]

Hap_15 ..........---.............................-
..................................... [160]

Hap_16 .......C..---T...........T..............C.-
T........C.....T...........T........C [160]

Hap_17 .......C..---T...........T..............C.-
T........C.....T...........T........C [160]

Hap_18 .......C..---T...........T................-
T........C.....T...........T........C [160]

Hap_19 .......C..---T...........T................-
T........C.....T...........T........C [160]

Hap_20 .......C..---T...........T................-
T........C.....T...........T........C [160]

Hap_21 .......C..---T...........T................-
T........C.....T...........T........C [160]

Hap_22 .......C..---T...........T................-
T........C.....T...........T......... [160]
```

Hap_23 ..........---T...........T................-
T........C.....T.....T..............C [160]

Hap_24 ..........---T.....T.....T...........C....-
T........C.....T...........T........C [160]

Hap_25 ..........---T.....T.....T...........C....-
T........C.....T...........T........C [160]

Hap_26 ..........---T.....T.....T...C.......C....-
T........C.....T...........T........C [160]

Hap_27 ..........---............T..............C.-
T..............T.....T.....T........C [160]

Hap_28 ..T.......---....T......AT.............TC.-
T..............T.....T.....T........C [160]

Hap_29 ..T.......---....T.......T..............C.-
T..............T.....T.....T........C [160]

Hap_30 .......C..---T...........T................-
T........C.....T...........T........C [160]

Hap_31 .......C..---T...........T................-
T........C.....T...........T........C [160]

Hap_32 .......C..---T...........T..............C.-
T........C.....T...........T........C [160]

Hap_33 .......C..---T...........T................-
T..............T...........T........C [160]

Hap_34 ..........---............T..............C.-
T..............T.....T.....T........C [160]

Hap_35 ..........---............T..............C.-
T..............T.....T.....T........C [160]

Hap_36 ..........---............T..............C.-
T..............T.....T.....T........C [160]

Hap_37 ..........---............T................-
T..............T.....T.....T........C [160]

Hap_1 AC---TCTAAACGTTGACACACGAGCATATTTTACTTCAGCC-
CAATAATCATTGCTATTCCCACTGGTGT---AGTTTT [240]

Hap_2 ..---.....................................-
............................---...... [240]

Hap_3 ..---.....................................-
............................---...... [240]

Hap_4 ..---.................................C...-
............................---...... [240]

Hap_5 ..---.....................................-
.........................G..---...... [240]

Hap_6 ..---.....................................-
............................---...... [240]

```
Hap_7  ..---....................................-
...........................---...... [240]
Hap_8  ..---....................................-
...........................---...... [240]
Hap_9  ..---....................................-
...........................---...... [240]
Hap_10 ..---....................................-
...........................---...... [240]
Hap_11 ..---....................................-
...........................---...... [240]
Hap_12 ..---....................................-
...........................---...... [240]
Hap_13 ..---....................................-
...........................---...... [240]
Hap_14 ..---....................................-
...........................---...... [240]
Hap_15 ..---....................................-
...........................---...... [240]
Hap_16 ..---C...................................-
........................C..---...... [240]
Hap_17 ..---C...................................-
........................C..---...... [240]
Hap_18 ..---C...................................-
.......T................C..---...... [240]
Hap_19 ..---C...................................-
.......T................C..---...... [240]
Hap_20 ..---C...................................-
........................C..---...... [240]
Hap_21 ..---C...................................-
........................C..---...... [240]
Hap_22 ..---C...................................-
........................C..---...... [240]
Hap_23 ..---C...................................-
........................C..---...... [240]
Hap_24 ..---C...................................-
........................C..---...... [240]
Hap_25 ..---C...................................-
........................C..---...... [240]
Hap_26 ..---C...................................-
........................C..---...... [240]
Hap_27 ..---C......................C............-
........................C..---...... [240]
```

Hap_28 ..---C.......................C............-
.........................C..---...... [240]

Hap_29 ..---C.......................C............-
.........................C..---...... [240]

Hap_30 ..---C....................................-
.........................C..---...... [240]

Hap_31 ..---C....................................-
.........................C..---...... [240]

Hap_32 ..---C....................................-
.........................C..---...... [240]

Hap_33 ..---C....................................-
.........................C..---...... [240]

Hap_34 ..---C.......................C............-
.........................C..---...... [240]

Hap_35 ..---C.......................C............-
.........................C..---...... [240]

Hap_36 ..---C.......................C............-
................C........C..---...... [240]

Hap_37 ..---C....................................-
.........................C..---...... [240]

Hap_1 ----TGGCTGGCCACCATGCACGGCGGTGTTAT------AGAAG-
TCCAATATTGTGAGCATT---CTTCATTTTCTTAT [320]

Hap_2 ----.............................------.....-
..................---.............. [320]

Hap_3 ----.............................------.....-
..................---.............. [320]

Hap_4 ----.............................------.....-
..................---.............. [320]

Hap_5 ----.............................------.....-
C..C.............C.---...T.......... [320]

Hap_6 ----.............................------.....-
..................---.............. [320]

Hap_7 ----.............................------.....-
..................---.............. [320]

Hap_8 ----.............................------.....-
..................---.............. [320]

Hap_9 ----.............................------.....-
..................---.............. [320]

Hap_10 ----.............................------.....-
..................---.............. [320]

Hap_11 ----.............................------.TG..-
..................---.............. [320]

```
Hap_12 ----............................------.TG..-
................C.---............. [320]

Hap_13 ----..A.........................------.TG..-
..................---............. [320]

Hap_14 ----..A.........................------.TG..-
..................---............. [320]

Hap_15 ----............................------.TG..-
..................---............. [320]

Hap_16 ----..AT....T..........T..CA.C..------.....-
.......C.A........---.....C....... [320]

Hap_17 ----..AT....T..........T..CA.C..------.....-
.......C.A........---.....C....... [320]

Hap_18 ----..AT....T..........T..CA.C..------.....-
.......C.A........---.....C....... [320]

Hap_19 ----..AT....T..........T..CA.C..------.....-
.......C.A........---.....C....... [320]

Hap_20 ----..AT....T..........T..CA.C..------.....-
.......C.A........---.....C....... [320]

Hap_21 ----..AT....T.......T..T..CA.C..------.....-
.......C.A........---.....C....... [320]

Hap_22 ----..AT....T..........T..CA.C..------.....-
.......C.A........---.....C....... [320]

Hap_23 ----..A.....T..............A.C..------.....-
.......C.A........---.....C....... [320]

Hap_24 ----..A.....T..........T...A....------.....-
.......C.A........---.....C....... [320]

Hap_25 ----..A.....T..........T..CA.C..------.....-
.......C.A........---.....C....... [320]

Hap_26 ----..A.....T..........T...A.C..------.....-
.......C.A........---.....C....... [320]

Hap_27 ----..A.....T..........T...A.C..------.....-
.......C.A........---.....C....... [320]

Hap_28 ----..A.....T..........T...A.C..------.....-
.......C.A........---.....C....... [320]

Hap_29 ----..A.....T..........T...A.C..------.....-
.......C.A........---.....C....... [320]

Hap_30 ----..AT....T.......T..T..CA.C..------.TG..-
.......C.A........---.....C....... [320]

Hap_31 ----..AT....T..........T..CA.C..------.TG..-
.......C.A........---T....C....... [320]

Hap_32 ----..AT....T..........T..CA.C..------.TG..-
.......C.A........---.....C....... [320]
```

Hap_33 ----..AT....T...........TT.CA.C..------.TG..-
.......C.A........---......C....... [320]

Hap_34 ----..A.....T...........T...A.C..------.TG..-
.......C.A........---......C....... [320]

Hap_35 ----..A.....T...........T...A.C..------.TG..-
.......C.A........---......C....... [320]

Hap_36 ----..A.....T...........T...A.C..------.TG..-
.......C.A........---......C....... [320]

Hap_37 ----..A.....T...........T...A.C..------.TG..-
.......C.A........---......C....... [320]

Hap_1 TCACCATC-----CCTAACCGGCATTGTTCTCGCCAACTCCTC-
ATCGATATTGTTCTTCACGACACCTACTACGTAGTC [400]

Hap_2 ....-----..............................-
.... [400]

Hap_3 ....-----..............................-
.... [400]

Hap_4 ....-----..............................-
.... [400]

Hap_5 ........-----..................T...........-
................................T... [400]

Hap_6 ....-----..............................-
.... [400]

Hap_7 ....-----..............................-
.... [400]

Hap_8 ....-----..............................-
.... [400]

Hap_9 ....-----..............................-
.... [400]

Hap_10 ....-----G.............................-
.... [400]

Hap_11 ....-----..............................-
.... [400]

Hap_12 ....-----..............................-
.... [400]

Hap_13 ....-----..............................-
.... [400]

Hap_14 ....-----..............................-
.... [400]

Hap_15 ....-----..............................-
.... [400]

Hap_16 .T......-----..................T...........-
....................T..............T [400]

```
Hap_17 .T......-----..................T...........-
....................T..............T [400]
Hap_18 .T......-----..................T...........-
....................T..............T [400]
Hap_19 .T......-----..................T...........-
..T.................T..............T [400]
Hap_20 .T......-----..................T...........-
....................T..A...........T [400]
Hap_21 .T......-----..................T...........-
....................T..............T [400]
Hap_22 .T......-----..................T...........-
....................T..............T [400]
Hap_23 .T......-----..................T...........-
.................T..T..............T [400]
Hap_24 .T......-----..................T...........-
....................T..............A [400]
Hap_25 .T......-----..................T...........-
....................T..............A [400]
Hap_26 .T......-----..................T...........-
....................T..............A [400]
Hap_27 .T......-----..................T...........-
.................T..............G..T [400]
Hap_28 .T......-----..................T...........-
..........G......T..............G..T [400]
Hap_29 .T......-----..................T...........-
.................T..............G..T [400]
Hap_30 .T......-----..................T...........-
....................T..............T [400]
Hap_31 .T......-----..................T...........-
....................T..............T [400]
Hap_32 .T......-----..................T...........-
....................T..............T [400]
Hap_33 .T......-----..................T...........-
....................T..............T [400]
Hap_34 .T......-----..................T...........-
.................T..............G..T [400]
Hap_35 .T......-----..................T...........-
.................T..............G..T [400]
Hap_36 .T......-----..................T...........-
.................T..............G..T [400]
Hap_37 .T......-----..................T...........-
.................T.................T [400]
```

Hap_1 GCCCATTTCCACTATGTATTATCAATAG---
CCGTTTTTGCTATCATAGCCGGATTTGTTCACTGATTCCC--------- [480]

Hap_2 ....---
....C...................................--------- [480]

Hap_3 ..................A.TA.T....---
....C...................................--------- [480]

Hap_4 ....---
....C...................................--------- [480]

Hap_5 ....---
.A..C...................................--------- [480]

Hap_6 ....---
.A..C...................................--------- [480]

Hap_7 ....T..........---
....C............A......................--------- [480]

Hap_8 ....---
....C...................................--------- [480]

Hap_9 ....---
....C...................................--------- [480]

Hap_10 ....---
....C...................................--------- [480]

Hap_11 ....---
....C...................................--------- [480]

Hap_12 ....---
....C...................................--------- [480]

Hap_13 ....C.........---
....C...................................--------- [480]

Hap_14 ....---
....C...................................--------- [480]

Hap_15 ....C.........---
....C...................................--------- [480]

Hap_16 ..T..C........C.............---
....C.......................C...........--------- [480]

Hap_17 ..T..C........C.............---
....C.......................C...........--------- [480]

Hap_18 ..T..C........C.............---
....C.......................C...........--------- [480]

Hap_19 ..T..C........C.............---
....C.......................C...........--------- [480]

Hap_20 ..T..C........C.............---
....C.......................C...........--------- [480]

Hap_21 ..T..C........C.............---
.T..C.......................C...........--------- [480]

```
Hap_22 ..T..C........C.............---
....C.......................C...........--------- [480]

Hap_23 ..T..C........C...C.........---
....C..............T.....C..C...........--------- [480]

Hap_24 ..T..C........C...C.........---
....C..............T.....C..C...........--------- [480]

Hap_25 ..T..C........C...C.........---
....C..............T.....C..C...........--------- [480]

Hap_26 ..T..C........C...C.........---
....C..............T.....C..C...........--------- [480]

Hap_27 ..T..C........C...C.........---
....C..............T.....C..C...........--------- [480]

Hap_28 ..T..C........C...C.........---
....C............A.T.....CC.C...........--------- [480]

Hap_29 ..T..C........C...C.........---
....C..............T.....CC.C...........--------- [480]

Hap_30 ..T..C........C.............---
.T..C.......................C...........--------- [480]

Hap_31 ..T..C........C.............---
.T..C.......................C...........--------- [480]

Hap_32 ..T..C........C.............---
....C.......................C...........--------- [480]

Hap_33 ..T..C........C.............---
....C.......................C...........--------- [480]

Hap_34 ..T..C........C...C.........---
....C..............T.....C..C...........--------- [480]

Hap_35 ..T..C........C...C.........---
....C..............T.....C..C...........--------- [480]

Hap_36 ..T..C........C...C.........---
....C..............T.....C..C...........--------- [480]

Hap_37 ..T..C........C...C.........---
....C..............T.....C..C...........--------- [480]


Hap_1  ---- [484]

Hap_2  ---- [484]

Hap_3  ---- [484]

Hap_4  ---- [484]

Hap_5  ---- [484]

Hap_6  ---- [484]

Hap_7  ---- [484]

Hap_8  ---- [484]
```

```
Hap_9  ---- [484]
Hap_10 ---- [484]
Hap_11 ---- [484]
Hap_12 ---- [484]
Hap_13 ---- [484]
Hap_14 ---- [484]
Hap_15 ---- [484]
Hap_16 ---- [484]
Hap_17 ---- [484]
Hap_18 ---- [484]
Hap_19 ---- [484]
Hap_20 ---- [484]
Hap_21 ---- [484]
Hap_22 ---- [484]
Hap_23 ---- [484]
Hap_24 ---- [484]
Hap_25 ---- [484]
Hap_26 ---- [484]
Hap_27 ---- [484]
Hap_28 ---- [484]
Hap_29 ---- [484]
Hap_30 ---- [484]
Hap_31 ---- [484]
Hap_32 ---- [484]
Hap_33 ---- [484]
Hap_34 ---- [484]
Hap_35 ---- [484]
Hap_36 ---- [484]
Hap_37 ---- [484]
```

;

end;

Characteristics of a microhabitat checklist (Inger's Habitat Code in Heyer *et al*. 1994; Zainudin *et al*. 2017; Zulkefli & Zainudin 2022).

Characteristic	Code	Acronym	Note
Habitat:	1	VA	Primary rain forest, hilly
Vegetation type	2	VB	Primary rain forest, flat
	3	VC	Deciduous dipterocarp
	4	VW	Peat swamp
	5	VK	Kerangas
	6	VRF	Riverine Forest
	7	VAgr	Agriculture
	8	VS	Marsh
	9	VE	Edge mixed dipterocarp forest (MDF)
	10	VF	Large clearing (camp, etc.)
	11	VG	Secondary growth, immature or regenerating forest
	12	VH	Gallery forest
	13	VJ	Selectively logged forest
	14	VR	Rubber or oil palm planting
	15	VT	Oak/chestnut montane forest
Microhabitat: Horizontal position	16	HPA	Permanent stream: in stream, actually in water
	17	HPB	Permanent stream: midstream on bar, rock or snag
	18	HPC	Permanent stream: on bank (distant to water),
	19	HPD	Intermittent stream: in stream, actually in water
	20	HPE	Intermittent stream, midstream on bar, rock or snag
	21	HPF	Intermittent stream, on bank (distant to water),
	22	HPG	Distant from any body of water, distance (m) to nearest stream
	23	HPH	In dried bed of intermittent stream
	24	HPJ	Temporary pond, in water

	25	HPK	Temporary pond, on bank, distance (m) from water
	26	HPL	Temporary pond, on vegetation
	27	HPM	Permanent stream, on exposed bed, distant from water
	28	HPN	Permanent pond
	29	HPP	Permanent swamp
	30	HPQ	On or in building
	31	HPR	Permanent pond, on bank, distance (m) to water
	32	HPS	Permanent swamp, in water
	33	HPT	Permanent stream, on vegetation
	34	HPU	Permanent drainage, in plantation, on bank
	35	HPV	Permanent drainage, in plantation, in water
Microhabitat	36	VPA	Under surface of soil: depth (cm)
Vertical position	37	VPB	In or under dead leaves
	38	VPC	Under rock, maximum dimension (cm) of rock
	39	VPD	Under log [diameter (cm) of log]
	40	VPE	In log [diameter (cm) of log]
	41	VPF	On surface of bare soil
	42	VPG	On surface of leaf litter or dead leaves
	43	VPH	On rock [maximum dimension (cm) of rock]
	44	VPJ	On log [diameter (cm) of log]
	45	VPK	On seedling or herbaceous plant (<1 m tall)
	46	VPL	On shrub or young sapling (plant,1–7 m):
			height (m) above ground
	47	VPM	On tree or large vine (plant >7 m) height (m) above ground or water, at breast height (DBH) for woody plant
	48	VPN	On dead stump height (m) above ground
	49	VPO	In crown of fallen dead shrub or tree height (m) above ground
	50	VPQ	On grass blade height (m) above ground
	51	VPP	In grass
Microhabitat:	52	SA	Leaf of plant maximum dimensions (cm) of leaf

Substrate	53	SB	Stem or branch of herbaceous plant
	54	SC	Twig or branch of woody plant, diameter (cm) of perch
	55	SD	Trunk of shrub or tree
	56	SE	In epiphyte
	57	SF	Under bark of log, stump or tree
	58	SG	Bank mud
	59	SH	Bank sand or gravel
	60	SJ	Bank rock

The notion is that for every frog encountered a single notation for each element will describe that microhabitat (Heyer *et al.* 1994).

The five elements that were recorded for each observation are as follows:

1. Date and time of observation (24 hour clock);
2. General location, vegetation type, and elevation;
3. Horizontal position, with reference to bodies of water, shade casting vegetation and shore. Each position needs to be qualified in detail;
4. Vertical position (In relation to terrestrial environment, vertical position refers to subsurface, at soil surface exposed, or in water. In deep rivers, vertical position is defined as depth.);
5. Substrate [usually refers as mineral soil, dead leaves, log, rock or vegetation. Each substrate often requires finer subdivision (Table 3.1)].

REFERENCES

Heyer W R, Donnelly M A, MacDiarmid R W, Hayek L A C and Foster M S. (1994). *Measuring and monitoring biological diversity: Standard methods for amphibian*. London: Smithsonian Institution Press.
